# Rapid and Efficient Gene Editing for Direct Transplantation of Naive Murine Cas9^+^ T Cells

**DOI:** 10.3389/fimmu.2021.683631

**Published:** 2021-07-21

**Authors:** Snigdha Majumder, Isabelle Jugovic, Domenica Saul, Luisa Bell, Nadine Hundhausen, Rishav Seal, Andreas Beilhack, Andreas Rosenwald, Dimitrios Mougiakakos, Friederike Berberich-Siebelt

**Affiliations:** ^1^ Institute of Pathology, University of Wuerzburg, Wuerzburg, Germany; ^2^ Department of Internal Medicine 5, Hematology and Oncology, Friedrich-Alexander University (FAU) of Erlangen-Nuremberg, Erlangen, Wuerzburg, Germany; ^3^ Department of Medicine II, Center for Interdisciplinary Clinical Research (IZKF), University Hospital Wuerzburg, Wuerzburg, Germany; ^4^ Comprehensive Cancer Centre Mainfranken, University of Wuerzburg, Wuerzburg, Germany; ^5^ Deutsches Zentrum für Immuntherapie (DZI), Friedrich-Alexander University (FAU) of Erlangen-Nuremberg, Erlangen, Germany

**Keywords:** CRISPR/Cas9, gRNA-only, GvHD, metabolism, NFAT, naive T-cell gene editing, T-cell transfer, IRF4

## Abstract

Gene editing of primary T cells is a difficult task. However, it is important for research and especially for clinical T-cell transfers. CRISPR/Cas9 is the most powerful gene-editing technique. It has to be applied to cells by either retroviral transduction or electroporation of ribonucleoprotein complexes. Only the latter is possible with resting T cells. Here, we make use of Cas9 transgenic mice and demonstrate nucleofection of pre-stimulated and, importantly, of naive CD3^+^ T cells with guideRNA only. This proved to be rapid and efficient with no need of further selection. In the mixture of Cas9^+^CD3^+^ T cells, CD4^+^ and CD8^+^ conventional as well as regulatory T cells were targeted concurrently. IL-7 supported survival and naivety *in vitro*, but T cells were also transplantable immediately after nucleofection and elicited their function like unprocessed T cells. Accordingly, metabolic reprogramming reached normal levels within days. In a major mismatch model of GvHD, not only ablation of NFATc1 and/or NFATc2, but also of the NFAT-target gene IRF4 in naïve primary murine Cas9^+^CD3^+^ T cells by gRNA-only nucleofection ameliorated GvHD. However, pre-activated murine T cells could not achieve long-term protection from GvHD upon single NFATc1 or NFATc2 knockout. This emphasizes the necessity of gene-editing and transferring unstimulated human T cells during allogenic hematopoietic stem cell transplantation.

## Introduction

Until today, immunological studies depend on mouse models, which provide a manipulable systemic approach. Hence, in order to understand cause and consequence of gene function in health and disease, multiple transgenic mice have been created. This is tedious, although modern *Clustered Regularly Interspaced Short Palindromic Repeats-associated protein 9 nuclease* (CRISPR/Cas9)-mediated techniques have improved the procedure enormously (1, 2). Some insights can also be gained if transgenic cells are analyzed *in vitro* or transferred to new mice. Lately, models of T-cell transfer additionally serve translational purposes. Prominent examples are Major histocompatibility complex (MHC, in humans also known as Human leukocyte antigen/HLA) mismatch models for graft-versus-host disease (GvHD), because they depict the inherent odds of allogeneic hematopoietic stem cell transplantation (allo-HCT). Any manipulation of T cells in GvHD models represents an idea to avoid or limit GvHD in the clinic.

To circumvent the creation of transgenic animals for such experiments, one could envisage an *in vitro* manipulation of primary T cells. However, in that case an efficient gene targeting is mandatory. Primary lymphocytes are difficult to transfect, which proves almost impossible for primary mouse T cells (3). For any success with electroporation or viral infection, usually activation is necessary. Yet, pre-activation directs the T cells towards a certain status before the transgene is expressed or an endogenous gene is inhibited and, importantly, before the T cells face the *in vivo* situation. Thus, the possibility for gene targeting of naive murine T cells is desirable, especially, when one wants to study genes involved in T-cell activation and differentiation. This, however, should be so effective that one can transfer the transgenic T cells without any further selection, enrichment and expansion.

Just recently, a technical breakthrough for efficient gene editing of primary T cells has been published (3, 4). The authors apply CRISPR/Cas9 technology. CRISPR/Cas9 is an RNA-guided endonuclease technique derived from a microbial defense system (5, 6). Target detection by base pairing ensures an extraordinary specificity. The RNA chaperone Cas9 consists of two nuclease domains, then generating a blunt-ended double strand break (DSB), the nucleation point for mutations. It allows the induction of ‘indel’ mutations caused by error-prone non-homologous end-joining repair (NHEJ). Indel mutations are insertions and deletions leading to frameshifts within a given coding region and consequently loss of the respective protein. In the technical paper mentioned (4), the authors transfect Cas9/target gene–specific CRISPR RNA (crRNA)/transactivating crRNA (tracrRNAs) ribonucleoproteins (RNPs) into human and mouse primary T cells. With this, they are even able to transduce non-activated T cells with high efficiency (3). Alternatively, pre-stimulated T cells from Cas9 transgenic mice can be mutated by guide (g) RNAs, the combination of crRNA and tracrRNA, which are delivered *via* retroviral transduction (7, 8). Since studies have shown that at least sole mRNA can be successfully electroporated into T cells (9), we attempted to make use of Cas9 transgenic mice (10) and to develop efficient gRNA-only delivery into naive T cells using a nucleofection technique.

If naive T cells shall be adoptively transferred after manipulation, Cas9-mediated knockout has to occur *in vivo*. In fact, T-cell receptor (TCR)-transgenic CD8^+^ T cells were nucleofected with RNPs to target surface proteins, immediately transplanted and mice infected to challenge the transgenic TCR. This resulted in efficient knockdown of targeted proteins (11). Nevertheless, for many mouse models it will be necessary to transfer more than one subtype of T cells to understand their interplay *in vivo* as well as to mimic their involvement in human diseases. For example, T cell-mediated GvHD obstructs allo-HCT, while T cell-mediated graft-versus-leukemia effect (GvL) limits relapses of leukemia, lymphoma or multiple myeloma. Here, the ratio of CD4/CD8 T-conventional cells (Tcon) and especially the dominant suppression of GvHD over GvL by Treg cells is decisive (12, 13). We had shown with a major mismatch model for allo-HCT and GvHD that co-transfer of total CD3^+^ T cells from NFAT-deficient mice shifts the balance towards CD8^+^ T cells and that Tregs function well in the absence of one or two NFAT members (14, 15). NFAT (nuclear factor of activated T-cells) is a transcription factor family, which is primarily activated by TCR signaling *via* the phosphatase calcineurin and therefore restrained by the calcineurin inhibitors cyclosporin A or tacrolimus applied to patients receiving allo-HCT (16, 17).

Here, we develop a method for sole nucleofection with a combination of RNAs, i.e. 1-3 chemically modified synthetic crRNAs and one tracrRNA into murine primary naïve T cells, isolated from Cas9-expressing mice. Such manipulated T cells survived rather well, preserved their naïve phenotype, were almost indistinguishable in their metabolic reprogramming, and importantly, presented with a high knockout efficiency. Our B6.*Cas9.Cd4cre*.*luc*.CD90.1 mice express Cas9 from double-positive CD4^+^CD8^+^ thymocytes on in all CD4^+^ – including CD4^+^CD25^+^ Tregs – and CD8^+^ T cells. In addition, due to the luciferase transgene, cells from these mice are trackable by bioluminescence after transfer and recognizable by the congenic marker CD90.1. Accordingly, we performed allo-HCT together with manipulated T cells to induce acute GvHD (aGvHD). Assuring, only gRNA-transfected and CRISPR/Cas9-edited primary naïve murine T cells behaved as if naïve T cells were isolated from NFAT-deficient mice and directly transplanted. With this, we provide an easy and cost-effective method to create transgenic T cells for any adoptive T-cell transfer model, but especially to study the needs and obstacles during allo-HCT.

## Material and Methods

### crRNA Design and gRNA Assembly

crRNAs were selected using DESKGEN or CHOPCHOP (*Irf4*) online platform. The target area was limited to the first ∼40 % of the coding sequence. Guides targeting common exons between isoforms with the highest on-target and off-target scores were selected. crRNAs were ordered from Integrated DNA Technologies in their proprietary Alt-R format. crRNA and Alt-R CRISPR-Cas9 tracrRNA (IDT) were mixed in equimolar concentration (10 µl each) in nuclease-free PCR tubes, heated at 95°C for 5 min and then cooled at RT for 10 min to anneal.

### BM and T Cell Isolation

BM cells were isolated by flushing femur and tibia bones of *Rag1* ^−/−^ mice with PBS containing 0.1 % BSA and passed through a 70-μm cell strainer. Spleens and lymph nodes were directly passed through a 70-μm cell strainer, washed with PBS containing 0.1% BSA and enriched with Mojosort Mouse CD3 T cell Negative Isolation kit (Biolegend) according to the manufacturer’s instructions (14). CD4^+^ or CD8^+^ T cells were isolated using negative Isolation kit (Biolegend) according to manufacturer’s instructions.

### T-Cell Culture and Stimulation

T cells were cultured in RPMI media with 10% FCS (Gibco), 2 mM l-alanyl-l-glutamine (GlutaMAX; Gibco), 1 mM sodium pyruvate, 0.1 mM nonessential amino acids, 55 µM β-mercaptoethanol, 100 U/ml penicillin, 100 µg/ml streptomycin, and 10 mM Hepes (Invitrogen). Purified T cells were stimulated with plate bound 5 µg/ml anti-CD3, and soluble 1 µg/ml anti-CD28 and 10 ng/ml IL-2 for 72h in 1 million cells per ml density. Naïve CD3^+^ T cells were either cultured with 5 ng/ml IL-7 for overnight before nucleofection or immediately nucleofected after purification.

### Nucleofection

T-cell culture media was pre-warmed in a CO_2_ incubator. T cells (2.5 million) were washed with Ca/Mg-free PBS to remove traces of FBS and resuspended in 100 µl of Ingenio Electroporation Solution. Cells were added on 3 µl (150 pmol) crRNA-tracrRNA duplex (gRNA) in a nuclease free tube, mixed gently by pipetting and incubated at RT for 2 min. When targeting two genes, 2 µl (100 pmol) of each gRNA was used. The cell RNA mix was then transferred in a cuvette and nucleofection was performed using Lonza Nucleofector™ IIb and X-001 preset program or using Lonza 4D Nucleofector and CM137 (for stimulated cells) or DS137 (for naïve T cells). Post nucleofection, pre-warmed media was added to the cells slowly and cells were carefully transferred to the 12-well plate. Plates were incubated in the CO_2_ incubator up to 3 days before transplantation into mice for a GvHD major mismatch model.

### Allogenic Hematopoietic Stem Cell Transplantation

BALB/c host mice (H-2^d^ CD90.2^+^) were conditioned by myeloablative total body irradiation (TBI) at a dose of 8.0 Gy using a Faxitron CP-160 X-ray system. Two hours after irradiation they were injected retro-orbitally with sex- and age matched 5 × 10^6^ C57BL/6 BM cells from *Rag1* ^-/-^ mice (H-2^b^ CD90.2^+^) with or without 2.5 × 10^6^ stimulated or 0.3× 10^6^ naïve CRISPR/Cas9-edited T cells from B6.*Cas9.Cd4cre*.*luc*.CD90.1 (H-2^b^ CD90.1^+^) mice. Mice were given antibiotic (Baytril, Bayer) for one week to avoid opportunistic infections. Transplanted mice were assessed daily for weight loss and clinical aGvHD score adapted from Cooke et al. (14, 18, 19).

### *In Vivo* and *Ex Vivo* Bioluminescence Imaging 

Mice were anesthetized by i.p. injection of 80 mg/kg body weight ketamine hydrochloride (Pfizer) and 16 mg/kg xylazine (CP Pharma). Together with anesthetics, mice were injected with 150 mg/kg D-luciferin (Biosynth). After 10 min, BLI signals of the anesthetized mice were recorded using an IVIS Spectrum Imaging system (Caliper Life Sciences). For *ex vivo* imaging of internal organs 6 d after allo-HCT, mice were injected with D-luciferin and sacrificed 10 min later. Internal organs were removed and subjected to BLI. All pictures were taken with a maximum of 5-min exposure time and analyzed with the Living Image 4.0 software (Caliper Life Sciences) (14, 19).

### Flow Cytometry Staining

Cells were washed once in FACS buffer (PBS containing 0.1% BSA) before blocking with anti-FcγRII/FcγRIII (2.4G2, BD Pharmingen). Staining of surface molecules (all Biolegend) was performed on ice using FITC-conjugated CD4 (RM4-5), CD8α (53-6.7), and CD90.1 (OX-7); PECy7-conjugated CD44 (IM7); PE-conjugated α4β7 (LPAM-1, DATK32), CD4 (RM4-5), CD8α (53-6.7), CD62L (MEL-14), CD44 and CD25 (PC61); APC conjugated CD90.1 (OX-7). Intracellular Foxp3 (FJK-16s, APC-conjugated; eBioscience), NFATc1 (anti-NFATc1 PE 7A6, Biolegend), IRF4 (3E4, APC or PB-conjugated; Biolegend) staining was performed using the Foxp3 staining kit (eBioscience) according to the manufacturer’s instructions. Antibodies (all Biolegend) for intracellular cytokine staining were APC-IFN-γ (XMG1.2), FITC-TNF-α (MP6-XT22) and PE-IL-2 (JES6-5H4). Cytokine detection was performed after a 6 h *in vitro* restimulation with 12-O-tetradecanoylphorbol-13-acetate (TPA; 10 ng/mL, Sigma) plus ionomycin (5 nM, Merck Biosciences) in the presence of GolgiStop and GolgiPlug (both BD Pharmingen) using the IC Fixation Buffer kit (eBioscience). Viable cells were detected with the Zombie Aqua™ Fixable Viability Kit (Biolegend). Data were acquired on a FACSCanto II (BD Biosciences) flow cytometer and analyzed with FlowJo software (Tree Star).

### Quantitative qRT-PCR

RNA was extracted from cultured cells using Trizol (Ambion/Life Technologies) followed by cDNA synthesis with the iScript II kit (BioRad). Quantitative RT-PCR was performed with an ABI Prism 770 light cycler with the appropriate primer pairs, Sequences available in **Table S2**.

### Indel Detection

Genomic DNA was extracted from cultured cells using Omega E.Z.N.A DNA/RNA Isolation kit. PCR (initial denaturation 95°C, 3 min, denaturation 95°C, 15 sec, annealing 55°C, 30 sec, elongation 72°C, 2 min, 40 cycles, final elongation 72°C, 10 min) was performed to amplify gRNA target regions using specific forward and reverse primer. The amplified product was gel purified and cloned in TA cloning vector kit (Promega) and transformed in DH5α E.coli (Invitrogen). Colonies were individually grown in LB broth and plasmid DNA was isolated. Presence of insert was confirmed by PCR. Clones were sequenced by Sanger sequencing using Hi-Di and ABI Prism Genetic Analyzer from Applied Biosystems. For performing ‘Tracking of Indels by Decomposition’ (TIDE), amplified target regions were gel purified and sequenced by Sanger sequencing. Sequencing files were uploaded in TIDE DESKGEN website along with gRNA sequence and analyzed using the TIDE tool. Primer sequences available in **Table S2**.

### Metabolism Study

*Compounds:* Glucose was purchased from Agilent/Seahorse Bioscience. Oligomycin, 2-deoxyglucose (2DG), Trifluoromethoxy carbonylcyanide phenylhydrazone (FCCP), antimycin A, and rotenone were purchased from Sigma Aldrich; Media for MST: XF Base Medium (Agilent Technologies), 2 mM sodium pyruvate, 10 mM Glucose, 2 mM L- Glutamine pH 7.4 +/- 0.05 (Sigma-Aldrich). Media for GST (All from Sigma-Aldrich): DMEM, 2 mM L- Glutamine pH 7.35 +/- 0.05. *Extracellular flux analysis:* Bioenergetics were determined as previously described (Böttcher et al., 2018). Briefly, one day prior measurements, Seahorse XFe96 culture plates (Agilent/Seahorse Bioscience) were coated with Corning™ Cell-Tak Cell and Tissue Adhesive (BD) with 0.1 M NaHCO_3_ (Sigma Aldrich) according to the manufacturer’s recommendations. A Seahorse XFe96 cartridge (Agilent/Seahorse Bioscience) was loaded with XF Calibrant solution (Agilent/Seahorse Bioscience) and incubated overnight at 37°C in a CO_2_-free atmosphere. The next day, cells were harvested from the culture, washed in assay-specific medium according to the manufacturer’s recommendations and viable cells were automatically counted on a Muse^®^ Cell Analyzer (Luminex Corp.). The cells were seeded at a density of 2.4 x 10^5^ T-cells per well. The ports of the Seahorse cartridge were loaded with appropriate dilutions of the following compounds (final concentrations in brackets): glucose (10 mM), oligomycin (1 µM), and 2DG (100 mM) for the GST and oligomycin (1 µM), FCCP (1.5 µM), and antimycin A/rotenone (3 µM each) for the MST. After sensor calibration, assays were run as detailed in the manufacturer’s manual by recording ECAR (extracellular acidification rate) and OCR (oxygen consumption rate). Metabolic parameters were obtained from the XF Wave software (Agilent/Seahorse Biosciences) and calculated using Microsoft Excel.

### Luciferase Reporter Assay

EL-4 cells were cultured in complete RPMI containing 5 % FCS (15). They were transiently transfected with an *Irf4* promoter luciferase-reporter construct. A 836 bp Irf4 fragment, generated by PCR with *Irf4*-Pr_s 5’ TTT GCT AGC CAT GAT TGA AAC TTT GGG G 3’ and *Irf4*-Ex1-Nco_a 5’ TTT CCA TGG TCC CAA GTT CAA GTG GTG 3’, was cloned into pGL3 *via* NheI/NcoI restriction sites, thereby matching the translational start site of IRF4 with that of luciferase. Plasmids expressing constitutive active HA-NFATc2 (20) or Flag-IRF4 were co-transfected by standard DEAE Dextran. 36 h post transfection, luciferase activity was measured from the cells that were left untreated or treated with TPA (10 ng/ml, Sigma), ionomycin (5 nM, Merck Biosciences) o/n and relative light units were corrected for the transfection efficacy based on total protein concentrations. Normalized mean values of at least 3 independent experiments are depicted in relative light units as fold activation over empty vector control.

### Immunoblot

T cells of spleen and LNs were harvested from *Nfatc1* ^caaA^ mice (21) crossed to dLckcre (22) and activated by 2.5 µg/ml ConA (C0412 Sigma). Whole cell extracts were resolved by 10% SDS-PAGE followed by immunoblotting (23). The primary antibodies used were rabbit anti-NFATc1/αA (IG-457, ImmunoGlobe), goat anti-IRF4 (sc-6059, Santa Cruz), and mouse anti-β-actin (C-4, Santa Cruz biotechnology).

### Statistical Analysis

Figures were prepared using GraphPad Prism 5 and Corel Draw software. Different groups were compared by Unpaired Student’s t test or Mann Whitney test using GraphPad Prism 5 software. Differences with p values of less than 0.05 were considered significant: *p<0.05; **p<0.005, and ***p<0.001. Replicates, as indicated, are individual mice or experiments.

## Results

### Nucleofection of gRNAs Into Cas9^+^ T Cells Is as Effective as That of RNPs in WT T Cells

**Figure 1:**
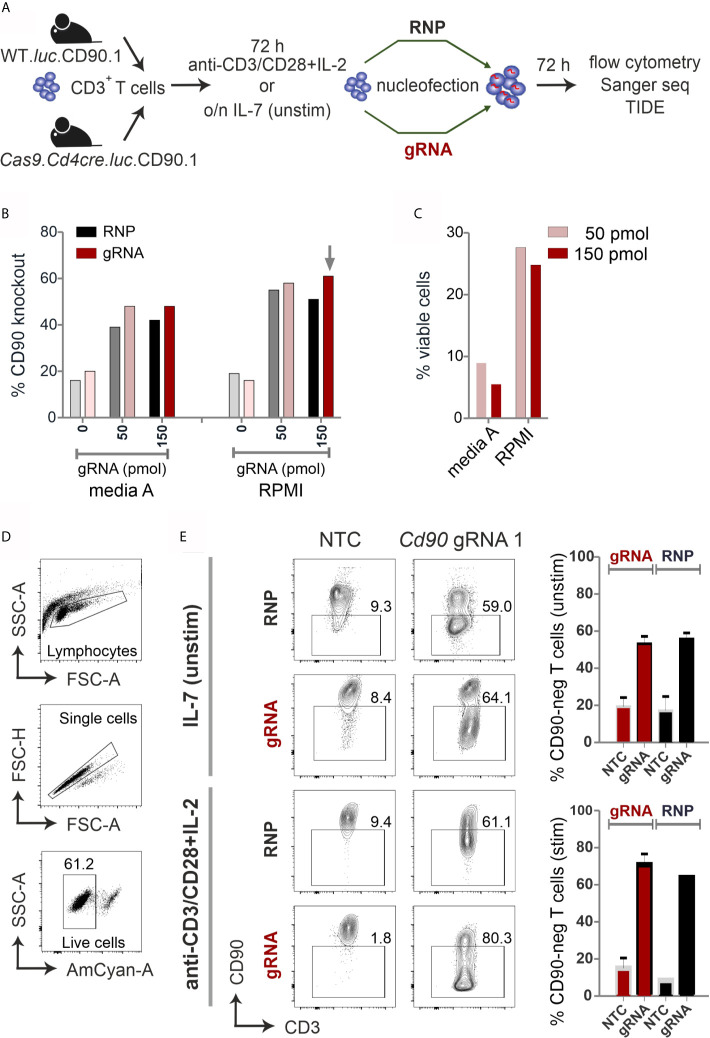
CRISPR/Cas9-mediated knockout is equally efficient with RNP nucleofection in WT or gRNA-only nucleofection in Cas9^+^CD3^+^ primary mouse T cells. **(A)** Flow chart of the CRISPR/Cas9 methods. **(B–E)** All analyses were performed by surface antibody staining and flow cytometry. **(B)** gRNA concentration optimization, comparison between Amaxa and RPMI media in resting CD3^+^ T cells. (n=1) **(C)** Viability of resting CD3^+^ T cells using gRNA nucleofection and different media. **(D)** Gating strategy and viability of pre-stimulated CD3^+^ T cells using gRNA nucleofection and RPMI. **(E)** Comparison of CD90 KO efficiency between RNP nucleofection in WT T cells and gRNA nucleofection in Cas9^+^ T cells, either resting (IL-7) or pre-stimulated CD3^+^ T cells, 3 days after nucleofection using IIb/X001 condition. Data are presented as mean ± SEM and representative of three independent experiments.

To eliminate the need of using recombinant Cas9 protein, we have bred *Rosa26-floxed STOP-Cas9* to *Cd4cre*-expressing mice (10, 24), which led to a deletion of the STOP cassette in CD4^+^ and CD8^+^ T cells. For subsequent transfer studies, these mice were crossed to L2G85.CD90.1 transgenic mice, which express firefly luciferase and CD90.1 as a congenic marker (14, 25). Thus, we generated B6.*Cas9.Cd4cre*.*luc*.CD90.1 (Cas9^+^) mice to isolate CD3^+^ T cells from spleen and lymph nodes. Now we could compare RNP and gRNA nucleofection in stimulated *vs* naive CD3^+^ T cells from wild type (WT) and Cas9^+^ mice, respectively (**Figure 1A**).

We prepared gRNA by combining chemically modified tracrRNA and crRNA (26). We used 50 and 150 pmol *Cd90* gRNA_1 (**Table S1**) to electroporate naïve mouse CD3^+^ T cells using Lonza Nucleofector IIb program X001. Seven days after nucleofection, flow cytometry analyses revealed around 60 % loss of surface protein expression and similar viability of around 25 % with complete RPMI media (**Figures 1B, C**). To investigate whether gRNA-only is as efficient as RNP-mediated knockout in naive mouse T cells collected from L2G85.CD90.1 mice (due to the lack of Cas9 expression referred to as WT), we prepared RNPs using 10 µg recombinant Cas9 protein and 150 pmol gRNA (3). RNP electroporation showed similar knockout efficiency in IL-7 pretreated naïve mouse T cells compared to gRNA electroporation (**Figure 1B**). Thus, we fixed our protocol for 150 pmol gRNA and RPMI, but verified this in an extended approach comparing RNP and gRNA-only nucleofection of unstimulated and by anti-CD3, anti-CD28 and IL-2 pre-stimulated CD3^+^ T cells from WT and Cas9^+^ mice for 72 hours. Viability was significantly enhanced in activated T cells (60 %; **Figure 1D**) as compared to naïve T cells (below 30%; **Figure 1C**). Nevertheless, no significant difference in knockout efficiency occurred between gRNA and RNP electroporation in neither unstimulated nor pre-stimulated T cells (**Figure 1E**). Collectively, we identified optimized conditions and demonstrated that gRNA delivery in Cas9^+^ naïve and pre-stimulated CD3^+^ T cells is as efficient as RNP nucleofection in WT cells.

### Two Genes Can Be Knocked Out Simultaneously in Stimulated Primary Mouse Cas9^+^CD3^+^ T Cells

We extended targeting to other genes encoding surface molecules, i.e. *Cd4* in isolated CD3^+^CD4^+^ T cells and *Cd8* in isolated CD3^+^CD8^+^ T cells (**Figure S1A**). Since efficacy and viability had been better with pre-stimulated T cells, we electroporated a combination of three gRNAs per gene this time using the 4D Nucleofector, CM137 program. After 72 h, CD90 and both co-receptors were lost with a consistent efficiency of above 90 %, all at once demonstrating that both T-cell subsets can be targeted equally well. Furthermore, different genes could be aimed at by a combination of three gRNAs per gene (**Table S1**) in the same cell, as proven by the concurrent loss of CD90 and CD8 in CD3^+^CD8^+^ T cells (**Figure S1B**). Overall, in both CD4^+^ and CD8^+^ T cells, only a slight decrease of the mean knockout efficiency per gene was observed when one additional gene was targeted simultaneously (**Figure S1C**).

### The Expression of Transcription Factor Nfatc1 Is Lost Upon gRNA-Only Nucleofection in Stimulated Murine Cas9^+^CD3^+^ T Cells

**Figure 2:**
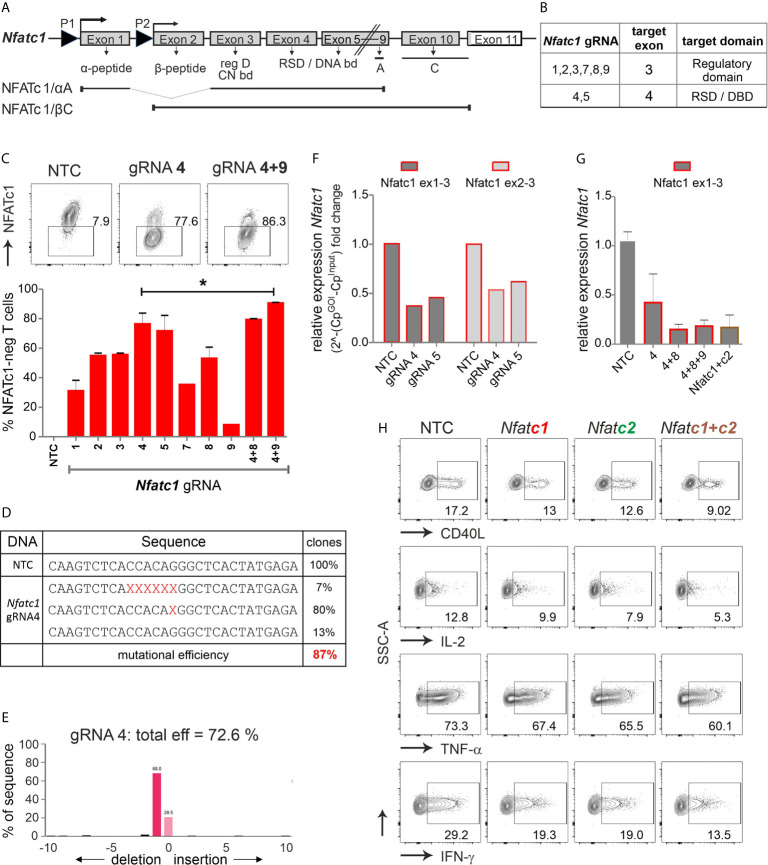
The combination of exon 3 and exon 4-targeting gRNAs efficiently erases *Nfatc1* in stimulated murine Cas9^+^CD3^+^ T cells. **(A)** Genomic structure of *Nfatc1* encoding six different isoforms due to two different promoters, of which P1 is inducible and P2 is constitutive, different splicing events and two non-depicted polyA sites. The most prominent isoforms NFATc1/αA and NFATc1/βC are indicated. The first common exon 3 encodes the regulatory domain, which includes calcineurin interaction and phosphorylation sites. Exon 4 is necessary for the expression of the Rel similarity domain, which enforces DNA binding. **(B)** Table with *Nfatc1* gRNAs and their target exons. **(C)** Variation in knockout efficiency between gRNAs specific for *Nfatc1* measured by intracellular flow cytometry for NFATc1. The efficiency increases by the use of two gRNAs per gene mean ± SEM, of three independent experiments, unpaired Student’s t test *P < 0.05. **(D)** Detection of indels in sequences of clones established after *Nfatc1* gRNA_4 nucleofection detected by Sanger sequencing, data are from one experiment. **(E)** Recognition of indels using TIDE. The mutational load was also calculated by TIDE. Of note, with variance R^2 =^ 0.92, 8 % were recognized as noise or large indels. **(F)** mRNA isolated from cells collected 72 hours post nucleofection. qRT-PCR with primers binding to exon 1 plus 3 or exon 2 plus 3 in *Nfatc1* RNA, i.e. 5’ of the gRNA_4 binding site. **(G)** qRT-PCR with primers binding to exon 1 plus 3 in *Nfatc1* cDNA after nucleofection of one, two or three *Nfatc1*-specific gRNAs, additionally with three *Nfatc1*-specific (4 + 8+9) and three *Nfatc2*-specific (1 + 2+3) gRNAs, mean + SEM, from two independent experiments. **(H)** Effect on target genes upon *Nfat*c1 and/or *Nfatc2* knockout. Flow cytometry of intracellular cytokines IL-2, TNF and IFN-γ as well as surface expression of CD40L. Data represent three independent experiments.

All NFAT proteins share a conserved core region composed of a DNA-binding ‘Rel-similarity domain’ and a less conserved N-terminal regulatory domain (**Figure 2A**, **Figure S2A**). Distinct NFAT family members and their isoforms have both redundant and specific functions, the latter most obvious for NFATc1 (19, 27, 28).

We had shown that co-transplantation of NFAT-deficient T cells as opposed to WT T cells ameliorates GvHD after HCT in a major mismatch model (14). At that time CD3^+^ T cells were gained from *Nfatc1* ^fl/fl^ *.Cd4cre* or *Nfatc2* ^-/-^ mice. For translational application, CRISPR/Cas9-mediated gene editing seemed feasible, but needed to be examined.

We designed several gRNAs for *Nfatc1* by the online tool DESKGEN (**Table S1**; **Figure 2B**). Comparing the efficiency of all individual gRNAs by single nucleofection determined *Nfatc1* gRNA_4, which binds to exon 4, as superior to all others (**Figure 2C**). The combination with exon 3-targeting gRNA_9 could enhance the degree of protein loss.

Direct evaluation of the mutational burden on DNA level - achieved by either cloning and sequencing or TIDE - revealed a similar high degree after gRNA-only_4 nucleofection (**Figures 2D, E**). In around 80 % of the cells, a single G nucleotide was deleted, expected to cause a frame shift (**Figure 2D**). About 7 % cells showed a deletion of six nucleotides as revealed by cloning PCR products of the target region in TA vector and Sanger sequencing. Although we could not detect any insertions using Sanger sequencing, TIDE data showed insertion of 2 to 10 nucleotides in very low frequencies of cells (**Figure 2E**). Of note, although during NHEJ most of the indels are in the length of a few nucleotides, it is still possible to have longer ones, not detectable by neither Sanger sequencing nor TIDE.

Interestingly, a reduction could be seen on mRNA level for both exon 1 and exon 2-containing isoforms even when the primers were chosen to bind 5’ of the seeding sequence of the employed gRNAs (**Figure 2F**). Loss of mRNA expression improved upon the combination of two or three gRNAs and did not alter when *Nfatc2* was additionally targeted (**Figure 2G**).

NFATc2, like NFATc1, comes in several isoforms, in which at least exon 5, 6, 7 are expressed in all. Resembling the strategy for *Nfatc1*, we had designed several *Nfatc2*-specific gRNAs, of which three gave the best results, i.e. two binding in exon 3 encoding most of the regulatory domain and one in exon 6 encoding part of the RSD (**Table S1**, **Figure S2B**). Here again, the knockout effect got stronger by combining *Nfatc2*-specific gRNAs specific for different exons (**Figure S2C**). Mutations caused by one gRNA were less efficient as in *Nfatc1*, but more complex (**Figures S2D, E**).

To evaluate the effect on NFAT-transactivated genes, we checked the expression of the cytokines IFN-γ, IL-2 and TNF-α as well as the surface molecule CD40L by flow cytometry. Although T cells had been stimulated for three days beforehand, which instigates instant NFAT activation, aiming at either NFATc1 or NFATc2 could still reduce target gene expression (**Figure 2H**). The combination of three *Nfatc1*-specific gRNAs with three *Nfatc2*-specific gRNAs elicited an augmented effect on NFAT response genes as compared to targeting NFATc1 or NFATc2 alone (**Figure 2H**). Taken together, we achieved above 85 % knockout efficiency of the transcription factor NFAT by applying gRNA-only nucleofection, which we confirmed on DNA, RNA and protein level. This had functional consequences for the manipulated T cells.

### NFAT-Reduced Pre-Stimulated Murine Cas9^+^CD3^+^ T Cells Expanded Poorly During HCT

**Figure 3:**
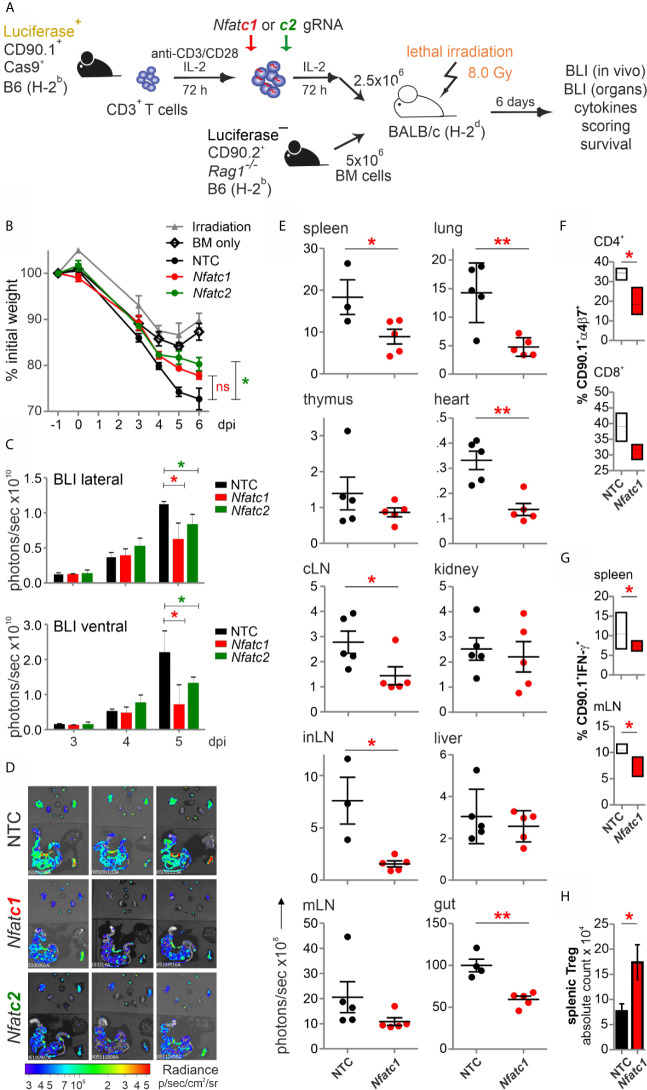
Knockout of NFATc1 or NFATc2 in pre-stimulated murine Cas9^+^CD3^+^ T cells limits signs of GvHD after co-transfer in a major mismatch model. **(A)** Experimental set up of sole NFAT-specific gRNA nucleofection and GvHD induction due to an H-2^b^ →H-2^d^ transfer with pre-stimulated Cas9^+^CD3^+^ T cells. **(B–H)** gRNAs used: *Nfatc1* gRNA 4 + 8+9, *Nfatc2* gRNA 1 + 2+3. **(B)** Weight changes of mice with NTC and NFAT-specific gRNA-nucleofected and co-transplanted Cas9^+^CD3^+^ T cells. Mice were evaluated every day pre and post transplantation and weight loss was calculated considering day-1 weight as 100 %. [ns, not significant] **(C)** Lateral and ventral view by BLI of living mice on day 3, 4, and 5 dpi with NTC, NFATc1 or NFATc2-ablated pre-stimulated Cas9^+^CD3^+^ T cells. Plotted are photons per second; mean + SD. **(D)** *Ex vivo* BLI images of lymphoid and non-lymphoid organs of the mice under **(C)** at 6 dpi; mean ± SD. **(E–H)** Analyses six days after GvHD induction with NTC and *Nfatc1* gRNA-nucleofected, pre-stimulated Cas9^+^CD3^+^ T cells; mean ± SD, unpaired Student’s t test (*p < 0.05, **p < 0.01), representative of two independent experiments. **(E)** Quantitation of *ex vivo* BLI analyses of lymphoid and non-lymphoid organs. **(F)** Staining of integrin α4β7 together with CD90.1, CD4 and CD8 followed by flow cytometry. **(G)** Intracellular staining of IFN-γ after surface staining for CD90.1, CD4 and CD8 followed by flow cytometry. **(H)** Intracellular staining of Foxp3 after surface staining for CD90.1, CD4 and CD25 followed by flow cytometry. [dpi, days post irradiation].

To explore whether NFAT ablation by CRISPR/Cas9 reduces the allo-reactivity like before (14), Cas9^+^CD3^+^luc^+^.CD90.1^+^.H-2^b^ T cells were nucleofected by three gRNAs targeting either *Nfatc1* or *Nfatc2* (**Figure 3A**). To achieve an enhanced degree of knockout (**Figure 1E**), we pre-stimulated the T cells before nucleofection. Subsequently, they were co-transferred with CD90.2^+^.H-2^b^ bone marrow (BM) from *Rag1* ^-/-^ mice into lethally irradiated CD90.2^+^.H-2^d^ BALB/c mice. All mice, which received T cells, lost weight continuously over six days, but less with a prior NFATc1 knockout and significantly less upon NFATc2 knockout (**Figure 3B**). Bioluminescence imaging (BLI) of the living mice over time revealed less proliferation and expansion of T cells, which had been NFAT-ablated *vs* transfected by non-targeting crRNA (NTC) (**Figure 3C**). This could be further corroborated *ex vivo* by BLI of all organs on day 6 (**Figure 3D**). Detailed comparison of lymphoid and non-lymphoid organs of mice with NTC *vs Nfatc1*-specific gRNA-nucleofected T cells documented a significant halt in the expansion of NFATc1 knockouts (**Figure 3E**). This included the gut, one of the prime target organs during GvHD. Accordingly, the gut homing receptor α4β7 integrin was less well expressed on NFATc1-ablated CD4^+^ and CD8^+^ T cells (**Figure 3F**). Additionally, those T cells produced fewer IFN-γ, the dominant cytokine during aGvHD (**Figure 3G**), while NFATc1 knockout Tregs had an advantage over NTC-transfected ones (**Figure 3H**). This supports our former data that Tregs are less dependent on NFAT proteins and that they function well, when one or two NFAT family members are missing (14, 15). In sum, with regard to allo-reactivity, ablating NFAT by gRNA-only in pre-stimulated Cas9^+^CD3^+^ T cells seemed to be equivalent to the loss achieved using T cells from knockout mice, as seen in our earlier work (14).

### Pre-Stimulation of T Cells Alters Their Behavior *In Vivo*

Unexpectedly, mice, which received NFATc1 or NFATc2-ablated T cells, were not protected over time and deceased like those with NTC-T cells (data not shown). One possibility was that the few cells without knockout outcompeted the NFAT-deficient ones. Therefore, we performed an experiment with NFATc1 and NFATc2-deficient cells gained from *Nfatc1* ^fl/fl^ *.Cd4cre* and *Nfatc2* ^-/-^ mice, respectively (14). We treated them according to our model (**Figure 3A**) and compared them to NTC and gRNA-nucleofected for NFATc1 plus NFATc2. Very different from our results using these same NFAT-deficient T cells without stimulation, pre-activated CD3^+^ T cells from either *Nfatc1* ^fl/fl^ *.Cd4cre* or *Nfatc2* ^-/-^ caused long-term weight loss and enhanced clinical scores beginning one week after transplantation in the major mismatch model of HCT (**Figure S3A**). The NFAT single-deficient T cells were responsible for a pre-mature death in comparison to NTC-nucleofected or NFATc1c2 double-deficient ones, created by gRNA nucleofection of Cas9^+^CD3^+^ T cells. With this, non-manipulated Cas9^+^CD3^+^ T cells overgrowing the factually nucleofected ones were unlikely to be the cause of loss of protection over time.

One remaining possibility was that the 3-day pre-stimulation period changed the (allo-) reactivity of T cells. Therefore, we adapted our protocol to pre-stimulating Cas9^+^CD3^+^ T cells for only 24 h before gRNA nucleofection and transplanting them without a major rest. In parallel, we verified the knockout on the level of *Nfatc1* and *Nfatc2* RNA (**Figure S3B**). We chose to transfer just as many T cells (1.2x10^6^) as we had done before with naive T cells from NFAT-deficient mice (14). Now we observed a constant benefit upon NFAT ablation (**Figure S3C**). Still, shortly pre-stimulated Cas9^+^ T cells did not behave equally when knocked out for NFATc1 *vs* NFATc2 since NFATc1 deficiency was even more effective than the DKO regarding GvHD scores. This was in contrast to the former data with naive T cells from NFAT-deficient mice. Despite that, knocking out NFATc1, NFATc2 or both by gRNA-only in shorter pre-stimulated and directly co-transplanted Cas9^+^CD3^+^ T cells protected mice from aGvHD, also obvious for skin GvHD on day 21 (**Figure S3D**).

### In Unstimulated T Cells, CRISPR Efficiency Depends on the Electroporator

**Figure 4:**
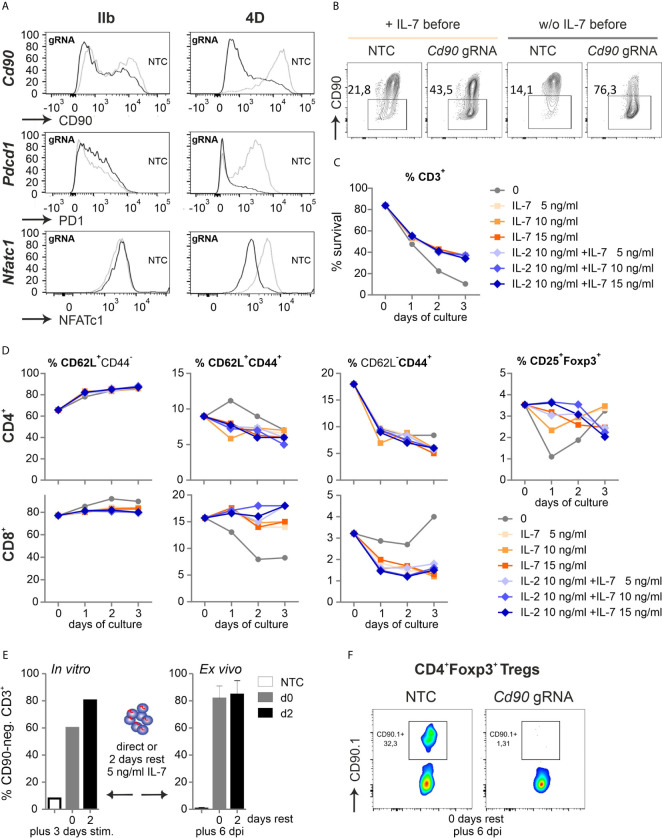
Naive Cas9^+^CD3^+^ T cells can be efficiently gene-ablated. **(A)** Comparison of two nucleofector versions for knocking out CD90 (gRNA 2 + 3), PD1 (gRNA 1 + 2) and NFATc1 (gRNA 4 + 8+9) by gRNA-only in resting Cas9^+^CD3^+^ T cells analyzed by flow cytometry after 3 d rest with IL-7 and 3 d of stimulation. **(B)** CD90 KO (gRNA 2 + 3) with and without o/n IL-7 pre-incubation, followed by nucleofection, 3 d rest with IL-7 and 3 d of stimulation. **(C, D)** Flow cytometric analyses over three days of resting Cas9^+^CD3^+^ T cells treated with 5, 10 and 15 ng/ml IL-7 in the absence or presence of 10 ng/ml IL-2 after NTC nucleofection. **(C)** Percentage of living CD3^+^ T cells analyzed by Zombie live-dead staining (mean ± SD). **(D)** Frequency of CD62L^+^CD44^-^ naive, CD62L^+^CD44^+^ central memory and CD62L^-^ CD44^+^ effector memory CD4^+^ and CD8^+^ T cells as well as CD4^+^CD25^+^Foxp3^+^ Tregs determined by surface and intracellular staining followed by flow cytometry. **(E)** CD90.1 staining and flow cytometry analysis of CD3^+^ T cells not rested or for 2 d with IL-7 post gRNA nucleofection (CD90 gRNA 1 + 2+3) followed by 3 d *in vitro* stimulation, n = 1; or transplanted and analyzed *ex vivo* 6 dpi; mean + SD. **(F)** CD90.1 expression on CD4^+^Foxp3^+^ T cells *ex vivo* 6 dpi.

Realizing the awkward performance of pre-stimulated CD3^+^ T cells after transfer, even not fully normal when pre-stimulation time was shortened to 24 h, we had to reconsider to manipulate and transfer naive T cells. We simply tried to nucleofect by making use of a more recent version of the electroporator. The technology and electrode material is different between the instruments (Aluminium for IIb and conductive polymer for 4D) and even preset programs are not comparable between them. CD90, PD1 and NFATc1 expression was evaluated after two gRNAs per gene had been transfected into naive Cas9^+^CD3^+^ T cells and all three proteins could now competently be reduced (**Figure 4A**, **Figure S4A**). Survival of naive T cells had been another issue (**Figure 1C**). First we re-evaluated the need of pre-culturing the naive Cas9^+^CD3^+^ T cells in IL-7 (3). No influence on survival could be observed by the addition of IL-7 for overnight rest, but undoubtedly, an improved knockout efficiency in the absence of IL-7 before nucleofection for three genes tested (*Cd90* in **Figure 4B**). Next, we evaluated how nucleofected cells could be supported *in vitro* before stimulation. Different concentrations of IL-7 were given alone or in combination with IL-2 over three days after nucleofection. In the presence of IL-7, nucleofected naive Cas9^+^CD3^+^ T cells survived better (**Figure 4C**). Here, any addition to 5 ng/ml IL-7 was not superior. Naivety – measured by CD62L and CD44 expression – was kept up, while central and effector memory occurrence was even counteracted by IL-7 in CD4^+^ and CD8^+^ T cells (**Figure 4D**). Tregs, which are highly sensitive *in vitro*, survived in sufficient frequencies, but surprisingly did not benefit from a further addition of IL-2.

To test whether IL-2 was functional, we repeated the experiment including IL-2 alone (**Figure S4**). Compared to IL-7 or IL-7 plus IL-2, IL-2 alone negatively affected the overall survival of CD3^+^ T cells, but could – in a known feedback loop *via* STAT5 activation – upregulate the high affinity receptor of IL-2, i.e. CD25 (**Figure S4B**). The percentage of Tregs was indeed supported by IL-2, although absolute Treg numbers did not differ between IL-2, IL-7 or double treatment (**Figure S4C**). Since IL-2 enforces central memory in CD4^+^ and effector memory in CD8^+^ T cells (**Figure S4D**), such treatment is not advisable if one wants naive T cells for analyses or transfer.

For *in vivo* experiments, many cells might be needed, but doubling the number of naive Cas9^+^CD3^+^ T cells per cuvette during nucleofection decreased the knockout efficiency, which was noticeable for CD90 and NFATc1 (**Figure S4E**). Lastly, we tested if gRNA-only-nucleofected naive Cas9^+^CD3^+^ T cells need to be rested *in vitro* to achieve the loss of target gene expression during transfer mouse models. After three days of *in vitro* stimulation, the 2-day period of rest appeared to enhance the number of gene-ablated cells (**Figure 4E**). On the other hand, cells, which were immediately transferred after nucleofection, exhibited the same high degree of 80 % knockout, when regained after six days as when they had been rested in IL-7 post nucleofection for two days prior to transplantation (**Figure 4E**). Since we nucleofected the mixture of resting CD3^+^ T cells, it was necessary to document whether the minor, but important subpopulation of Tregs got targeted together with all Tcon. Indeed, the knockout efficiency was high in Tregs as well, when measured directly transplanted and six days after GvHD induction (**Figure 4F**). Thus, all Cas9^+^CD3^+^ T cells can be efficiently gene-targeted by gRNA-only without pre-stimulation if the right nucleofector is used, they are supported directly by the *in vivo*-situation or by some IL-7 after nucleofection and before activation *in vitro*.

### The Procedure of Nucleofection Only Minimally Affects the Metabolism of Cas9^+^CD3^+^ T Cells

From their naïve to effector function, T cells undergo metabolic reprogramming and shift from oxidative phosphorylation (OXPHOS) towards aerobic glycolysis. The co-secretion of protons and lactate during aerobic glycolysis results in the acidification of the media, which can be measured as the extracellular acidification rate (ECAR) in the Glycolysis Stress Test (GST). The Mitochondrial Stress Test (MST) is based on changes of the oxygen consumption rate (OCR) that is indicative for OXPHOS.

**Figure 5:**
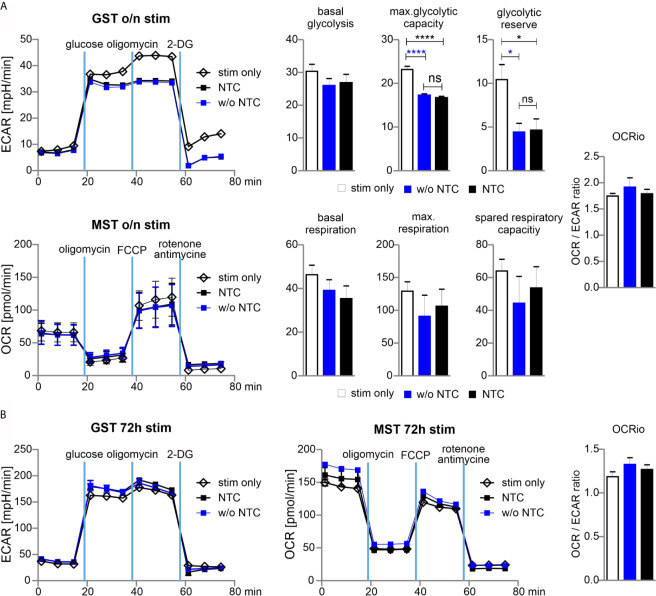
Glycolytic reserve has been compromised upon electroporation of T cells. **(A)** Naive mouse Cas9^+^CD3^+^ T cells were nucleofected followed by overnight stimulation with anti-CD3/CD28 and IL-2. Metabolic status was analyzed performing a GST and MST on a the XFe96 Seahorse metabolic flux analyzer. ECAR was measured at baseline, in response to glucose to calculate basal glycolysis, after oligomycin injection for max. glycolytic capacity and reserve, and after 2-DG injection for non-glycolytic acidification. Basal glycolysis = glucose - baseline; glycolytic capacity = oligomycin - baseline; glycolytic reserve = capacity - glycolysis. Oxygen consumption rate (OCR) was measured under basal condition followed by sequential injection of oligomycin, FCCP, and rotenone together with antimycin A to shut down mitochondrial respiration (values represent non-mitochondrial respiration). Basal respiration = baseline - non-mitochondrial respiration; maximal respiration = FCCP - non-mitochondrial respiration; spare respiratory capacity = maximal respiration - basal respiration. Student’s two-tailed *t*-test (*p < 0.05, ****p < 0.001)*;* mean ± SD, data represents two independent experiments. [ns, not significant] **(B)** Naive mouse Cas9^+^CD3^+^ T cells were nucleofected followed by 72 hours stimulation with CD3/CD28 and IL-2. Metabolic status was determined as for **(A)**. Student’s two-tailed *t*-test (*p < 0.05, ****p < 0.001)*;* mean ± SD; of two independent experiments.

In order to determine whether nucleofection of naive Cas9^+^CD3^+^ T cells influences their metabolic plasticity during activation, we performed metabolic flux analyses in cells, which underwent nucleofection with or without NTC in comparison to stimulated cells. As anticipated, without stimulation, ECAR and OCR activity was hardly detectable (data not shown) in T cells, while overnight (15 h) stimulation revealed substantial glycolytic and OXPHOS activity. Nucleofection, irrespective of incorporated RNA, affected both glycolysis and OXPHOS. The strongest effects were observed for maximal glycolytic capacity and glycolytic reserve, while the basal OCR/ECAR ratio was not skewed suggesting a balanced effect on the basal bioenergetic phenotype (**Figure 5A**). Strikingly, continuously (for 72 h) stimulated T cells did not display any differences in terms of their metabolic profile (**Figure 5B**). The latter observation indicates that nucleofection of naive Cas9^+^CD3^+^ T cells might stress them (metabolically) only transiently and that upon a short recovery period T cells preserve their full capacity to meet their basic bioenergetic demands, but also to adapt to increased demands through upregulation of aerobic glycolysis or OXPHOS.

### Naive NFAT-Targeted Cas9^+^CD3^+^ T Cells Do Not Cause Severe GvHD

**Figure 6:**
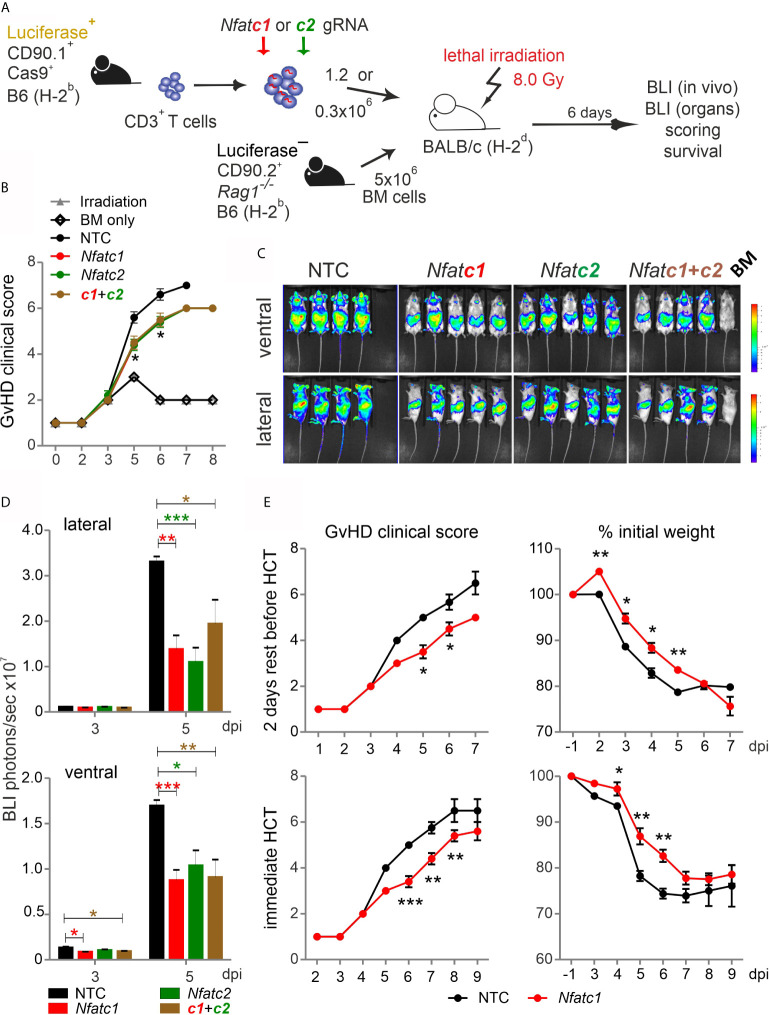
*Nfatc1* ^-/-^ ‘CRISPR’ed unstimulated murine Cas9^+^CD3^+^ T cells ameliorate aGvHD. **(A)** Experimental set up of sole NFAT-specific gRNA nucleofection and GvHD induction due to an H-2^b^ → H-2^d^ transfer with naive Cas9^+^CD3^+^ T cells. **(B–E)** gRNA used: *Nfatc1* gRNA 4 + 8+9, *Nfatc2* gRNA 1 + 2+3. **(B–D)** Naive Cas9^+^CD3^+^ T cells were nucleofected with NTC, *Nfatc1*, *Nfatc2*, and *Nfatc1* plus *Nfatc2* targeting gRNAs and 1.2x10^6^ cells transplanted immediately thereafter. **(B)** Clinical scores of GvHD-induced mice determined daily for 8 days. **(C)** Ventral and lateral in *vivo* BLI at 5 dpi. Data represent two independent experiments. **(D)** Quantitation and statistical analyses of BLI of living mice in lateral and ventral view on 3 and 5 dpi. Plotted are photons per second. Student’s two-tailed *t*-test (*p < 0.05, **p < 0.005, ***p < 0.001)*;* mean +SD. **(E)** Naive Cas9^+^CD3^+^ T cells were nucleofected by NTC or *Nfatc1* targeting gRNAs and 0.3x10^6^ cells transplanted after 2 days of rest in comparison to immediately. Clinical scores and weight loss were assessed daily over the indicated period. Student’s two-tailed *t*-test (*p < 0.05, **p < 0.005, ***p < 0.001)*;* Mean ± SD.

Pre-stimulated and nucleofected Cas9^+^CD3^+^ T cells were able to cause GvHD in a major mismatch model, during which NFAT deficiency reduced T-cell expansion and proliferation (**Figure 3**). However, the clinical score was untypically low and NFAT single-ablated T cells could not protect over time (**Figure S3**). Now we co-transplanted naive Cas9^+^CD3^+^ T cells with BM cells (**Figure 6A**). The clinical scores doubled, demonstrating the undisturbed power of naive T cells (**Figure 6B**). Knockout of NFATc1, NFATc2 or both reduced the clinical scores. Accordingly, NFAT-ablated cells - measured by *in vivo* BLI – expanded significantly less (**Figures 6C, D**).

Next, we analyzed whether it would be advisable to rest naive, NFAT-ablated Cas9^+^CD3^+^ T cells in IL-7 before transfer. We transferred the same number of BM cells, but a substantially downgraded number of T cells, still causing a clinical score above 6 (**Figure 6E**). Two days of rest enabled the T cells to transmit the high GvHD score earlier, but their potential for induction of clinical scores and weight loss were alike irrespective of rest or immediate transfer after nucleofection. Knockout of NFATc1 by gRNA-only in naive Cas9^+^CD3^+^ T cells limited GvHD symptoms significantly in both settings (**Figure 6E**). We conclude that gRNA-only nucleofection of unstimulated Cas9^+^CD3^+^ T cells not only leads to efficient gene editing, but preserves their functional abilities upon allo-HCT.

### Transfer of NFAT-Ablated Naive T Cells Protect Mice From GvHD Over Time

**Figure 7:**
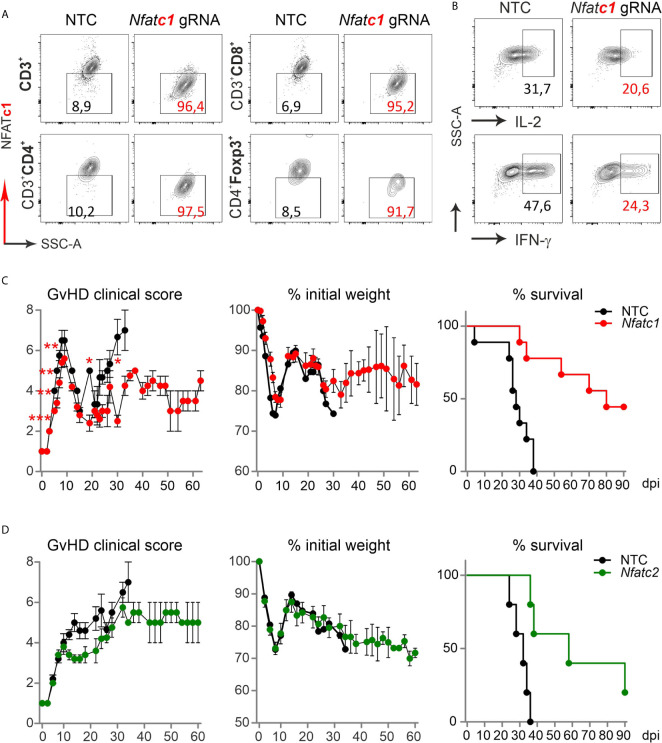
*Nfatc1* ^-/-^ and *Nfatc2* ^-/-^ ‘CRISPR’ed unstimulated murine Cas9^+^CD3^+^ T cells protect over time and prolong survival. **(A, B)** Naïve Cas9^+^CD3^+^ T cells were nucleofected by NTC or *Nfatc1*-targeting gRNAs and knockout efficiency was assessed by surface and intracellular flow cytometry after 2 days of IL-7 rest and 3 days of stimulation in *vitro*. **(A)** Detection of NFATc1 in CD4^+^ and CD8^+^ as well as CD4^+^Foxp3^+^ T cells. **(B)** Intracellular IL-2 and IFN-γ staining in *Nfatc1* gRNA-nucleofected naïve Cas9^+^CD3^+^ T cells. **(C, D)** Naive Cas9^+^CD3^+^ T cells were nucleofected by NTC or NFAT-specific gRNAs (*Nfatc1* gRNA 4 + 8+9, *Nfatc2* gRNA 1 + 2+3) and 0.3x10^6^ cells transplanted directly thereafter (H-2^b^ → H-2^d^ transfer). Clinical scores and weight loss were determined over 60 days, whereas survival over 90 days. Student’s two-tailed *t*-test (*p < 0.05, **p < 0.005, ***p < 0.001)*;* mean ± SEM, n≥5. Data represent two independent experiments.

To evaluate if the direct transfer of NFAT-deficient T cells created by gRNA-only nucleofection in naive Cas9^+^CD3^+^ T cells impinged long-term protection from severe GvHD, we once again knocked out NFATc1. NFATc1 ablation was verified in the total CD3^+^ T cell population and individually in CD4^+^ and CD8^+^ Tcon as well as CD4^+^Foxp3^+^ Tregs, each time in comparison to NTC nucleofection, by intracellular flow cytometry (**Figure 7A**). When we rested some of those cells in IL-7 for two days, stimulated them with anti-CD3/28+IL-2 and restimulated by PMA/Ionomycin for 5 h *in vitro*, IL-2 and IFN-γ expression was compromised due to NFATc1 deficiency (**Figure 7B**). *In vivo*, different from the transfer with pre-stimulated T cells, clinical scores as well as weight loss remained less severe over time in comparison to the transfer of naive WT Cas9^+^CD3^+^ T cells (**Figure 7C**). Accordingly, all mice, which received NTC-nucleofected Cas9^+^CD3^+^ T cells, had died by 35 days, while half of all mice getting NFATc1 knockout T cells were still alive after 90 days. Similarly, NFATc2 ablation in naive Cas9^+^CD3^+^ T cells and their subsequent transfer limited the degree of GvHD stably over time (**Figure 7D**). With this, naive T cells behaved the same in our major mismatch model irrespective whether they were gathered from NFAT-deficient mice (14) or whether they were knocked out *in vitro* by gRNA-only nucleofection of Cas9^+^CD3^+^ T cells.

### Ablation of the NFAT Target Gene *Irf4* in Donor T Cells Ameliorates GvHD

If T cells are not stimulated before allo-HCT, NFAT target genes are not yet trans-activated. This might be one functional difference between transplanted naive and activated T cells. Besides effector molecules like CD40L or cytokines, NFAT induces transcription factors, thereby influencing gene expression in an extensive manner. As we found NFAT to upregulate and cooperate with IRF4 (29, 30), we determined whether IRF4 is a direct target gene. Both NFATc1 and NFATc2 are bound to the immediate upstream region of *Irf4* in ChIPseq experiments of CD8^+^ T cells (**Figure S5A**) (31, 32). Accordingly, constitutive active NFATc2 transactivated the *Irf4* promoter in a reporter assay (**Figure S5B**), while activation of T cells from *Nfatc1* ^caaA^.dLckcre mice, which express constitutive active NFATc1/αA in post-thymic T cells, had a strong positive impact on IRF4 protein levels (**Figure S5C**).

**Figure 8:**
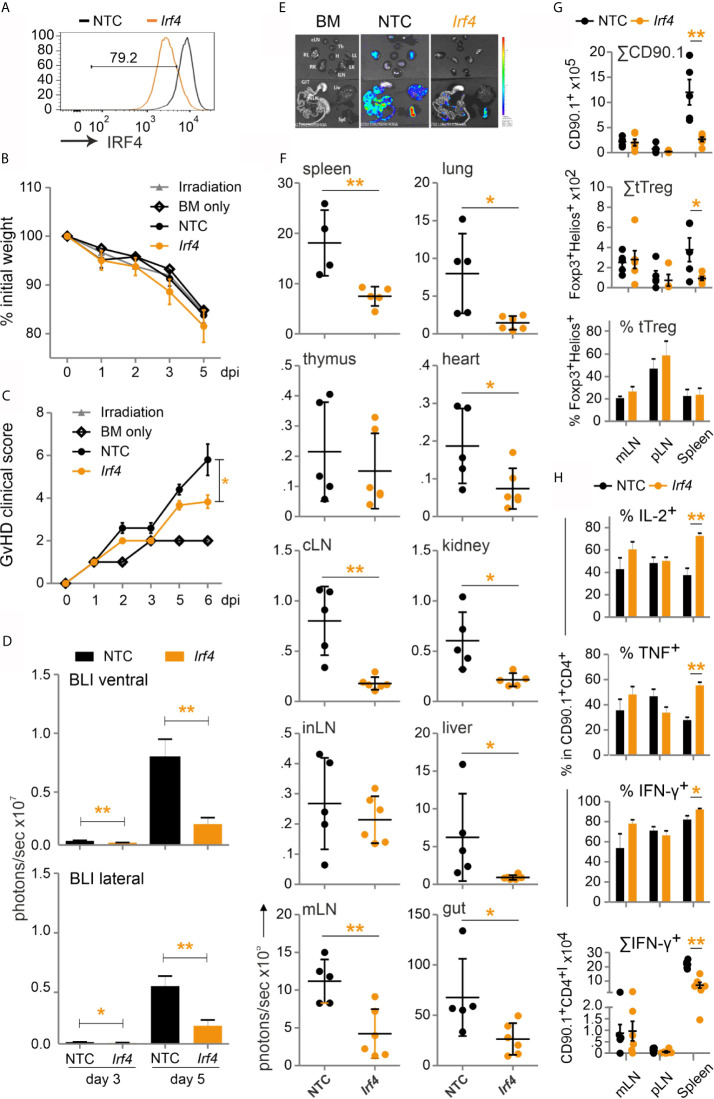
*Irf4* ^-/-^ CRISPR’ed unstimulated murine Cas9^+^CD3^+^ T cells ameliorate GvHD despite enhanced Th1 differentiation. **(A–H)** Naïve Cas9^+^CD3^+^ T cells were nucleofected with either NTC or combination of three gRNAs targeting *Irf4.* **(A)** IRF4 knockout efficiency analyzed in Cas9^+^CD3^+^ T cells by intracellular staining and flow cytometry post 2 days rest with IL-7 and 3 days of stimulation. **(B–H)** GvHD induction due to an H-2b →H-2d transfer with naïve Cas9^+^CD3^+^ T cells. Mann Whitney test (*p < 0.05, **p < 0.005). Data represent mean ± SD from one experiment with n≥5 mice per group. **(B)** Weight was measured post transplantation up to dpi 5 and percentage of weight loss was calculated considering d0-weight as 100%. **(C)** Clinical score of GvHD-induced mice determined daily for 6 days. **(D)** Quantitation of ventral and lateral *in vivo* BLI at 3 and 5 dpi. **(E)** *Ex vivo* BLI images of lymphoid and non-lymphoid organs at 6 dpi. **(F)** Quantitation of *ex vivo* BLI analyses of lymphoid and non-lymphoid organs at 6 dpi. **(G)** Absolute count of CD90.1^+^ donor T cells and CD90.1^+^CD4^+^CD25^+^Foxp3^+^ donor T cells by flow cytometry. Percentage of Helios^+^ tTreg within CD90.1^+^CD4^+^CD25^+^Foxp3^+^ donor T cells by intracellular flow cytometry. **(H)** Frequency of IL-2^+^, TNF^+^ and IFN-γ^+^ within CD90.1^+^CD4^+^ donor T cells determined by intracellular staining and flow cytometry as well as absolute count of CD90.1^+^CD4^+^ IFN-γ^+^ donor T cells.

This prompted us to test our established method of CRISPR/Cas9 editing by gRNA-only nucleofection with this NFAT target gene for allo-HCT. gRNAs for exon 1 and exon 6 were tested in different combinations. The combination of three exon-1-specific gRNAs for nucleofection of naive Cas9^+^CD3^+^CD90.1^+^ T cells achieved 80 % IRF4-negative T cells after 2 days rest with IL-7 and 3 days of stimulation *in vitro* (**Figure 8A**). Editing of *Irf4* and direct transfer in conjunction with allo-HCT did not prevent weight loss, but reduced the clinical score significantly (**Figures 8B, C**). Proliferation and expansion of transplanted T cells was extensively impaired (**Figure S5D**, **Figure 8D**). This could also be observed *ex vivo* in individual organs (**Figures 8E, F**). Accordingly, the absolute number of *Irf4* ^-/-^ CD90.1^+^ T cells – including that of tTregs – was less compared to NTC-nucleofected T cells (**Figure 8G**). However, the frequency of *Irf4* ^-/-^ Tregs was preserved within the transplanted T-cell fraction (**Figure 8G**). This might be due to relatively more IL-2 and TNF-expressing CD4^+^ and CD8^+^ splenic T cells (**Figure 8H**, **Figure S5E**), which support Tregs *via* CD25, i.e. the high-affinity IL-2R, and TNFR2. On the other hand, we observed an enhanced Th1 phenotype, i.e. IFN-γ and again TNF production, caused by IRF4 ablation in CD4^+^ and CD8^+^ T cells (**Figure 8H**, **Figure S5E**). Nevertheless, the absolute numbers of cytokine-expressing as well as granzyme B and perforin-positive CD4^+^ and CD8^+^ *Irf4* ^-/-^ T cells were contracted significantly in comparison to NTC-nucleofected T cells (**Figure 8H**, **Figures S5F, G**). In sum, despite the shift towards an unfavorable Th1 differentiation, deletion of the NFAT target gene IRF4 in co-transplanted naive T cells during allo-HCT protected from severe GvHD.

## Discussion

We present an effective CRISPR/Cas9-based method to edit genomes in primary murine T cells. If Cas9 transgenic mice are available, gRNA-only nucleofection is sufficient in pre-activated and even in naive Cas9^+^CD3^+^ T cells to achieve at least 80 % knockout. More than one gRNA per gene increased the degree of knockout, especially when targeting different exons encoding different protein domains. By nucleofection of multiple gRNAs, it is possible to ablate several genes concurrently with almost unchanged effectiveness per gene. Importantly, nucleofected resting Cas9^+^CD3^+^ T cells could be transferred to mice without any further treatment or rest, acquired their knockout *in vivo*, but otherwise behaved like transplanted naive CD3^+^ T cells. Further requirement fulfilled was that nucleofection only transiently affected the metabolism of Cas9^+^CD3^+^ T cells.

Primary mouse T cells are usually not easily genome-edited by non-viral methods, wherefore transgenes or shRNAs are mostly transferred retro- or lentivirally into pre-activated cells, often with a subsequent selection by antibiotics or flow cytometric sorting. Researchers have created Cas9 transgenic mice (8, 10). To manipulate their T cells, however, gRNAs are still introduced per retroviral transduction (7, 8). Apart from the fact that this is more tedious and time consuming than electroporation, retroviral backbones could cause immunogenicity and toxicity (33). Of note, T cells have to be activated before retroviral infection. Thus, overall transfection of transgenic Cas9^+^ cells by electroporation appears advantageous.

Nonetheless, electroporation entails other challenges. Electroporation of cells can be irreversible, when it disrupts the plasma membrane, causing loss of cell homeostasis and leading to cell death. On top, applied electric fields, even if they are transient, can reach the mitochondrion, which harbors the electron transport chain and can release cytochrome C, which would affect the metabolic capacities and again induce cell death (34). Thus, conditions have to be acquired, which allow the cells to fully recover from the transient perturbation. In fact, we did not observe any stress on mitochondria, in line with quiescence and survival of the manipulated T cells. Metabolic competence including a shift from OXPHOS towards aerobic glycolysis upon stimulation is a prerequisite for proper T cell function (35). In addition, although Tregs are known to rather utilize OXPHOS to exert their suppressive activity, they also rely on glycolysis for proliferation and migration (36). Monitoring T-cell bioenergetics after *in vitro* stimulation revealed a preserved full metabolic capacity for all T-cell subtypes three days after nucleofection. Induced stress on preferentially glycolytic reserve capacities was apparent only transiently after the intervention. Overall, we found conditions - with the right nucleofector and program - which ensure good survival rates of resting Tcon as well as Tregs allowing them to respond with unperturbed proliferation and metabolic reprogramming.

It surely makes a difference whether T cells lose a certain gene before they are activated or thereafter. For example, NFAT proteins transmit TCR signals, i.e. antigen recognition, which leads to a plethora of transactivated genes (16, 37, 38). First identified was the positive regulation of cytokine expression like that of IL-2 and IFN-γ. Not only IFN-γ, but already IL-2 influences immune cell differentiation (39). We recently showed that an enhanced amount of IL-2 – due to the dominance of NFATc1/αA – at the beginning of activation shifts the immune response to a more tolerogenic one, although upregulation of IL-2 is transient (19). This might be one reason, why only naive T cells, but not effector cells play a major role in acute GvHD in mice and men (40–42). At least our data gathered after the transfer of pre-activated/effector T cells in a major mismatch model confirmed this notion - and made it necessary to search for the right condition to manipulate naive T cells. Such data about the alloreactivity of naive *vs* pre-stimulated T cells stirred clinical studies and naive T cells were depleted by anti-CD45RA from the HCT grafts (43). It failed, possibly because CD45RA^+^ Tregs were excluded as well, while Tregs are needed to limit acute GvHD (44). This emphasizes the need of murine transfer models, in which more than one subtype of immune cells is studied. With respect to gene editing by CRISPR/Cas9 and T-cell transfer models, it highlights attempts like ours, in which CD3^+^ T cells instead of one T-cell subtype is edited and transplanted for GvHD. In this context, it is noteworthy that CD4^+^ and CD8^+^ Tcon as well as CD4^+^CD25^+^Foxp3^+^ Tregs were all efficiently gene-ablated in the mixture of Cas9^+^CD3^+^ T cells.

Since all T-cell subtypes are all edited equally well, this allows the approach to be used in wide-ranging scenarios. For hard to isolate subtypes or subtypes which differentiate *in vivo* after transfer, Cas9 transgenic mice can be bred to different Cre lines (for example to *Il21cre* for follicular T-helper (T_FH_) cells). Total CD3^+^ T cells will be nucleofected with gRNA and transplanted in recipient mice. Gene editing, however, will occur exclusively upon subtype-specific Cre expression. This definitely is an important application, which is not possible using the RNP method.

Therefore, Cas9^+^ mice, especially when they are already inbred as B6.*Cas9.Cd4cre*.*luc*.CD90.1 (or other Cre lines), are a suitable tool for comfortable subsequent studies. Although we avoided a repetition of GvL experiments with NFAT single-ablated T cells (14), successive experiments involving other genes or varying the GvHD model (acute GvHD due to minor mismatch or chronic GvHD) could easily include this aspect. In our context, we might want to knockout further NFAT target genes – additionally and in parallel – to test if the ‘NFAT phenotype’ is due to a certain gene’s altered expression. The limited study with the ablated NFAT target gene *Irf4* demonstrated already that absence of IRF4, which is dominantly required for Th2, Th17 and T_FH_ cell differentiation (45), provokes a disadvantageous Th1 phenotype under GvHD-inducing conditions. This is in contrast to allo-HCT with NFAT-deficient T cells, which can also implement specific T-helper characteristics (46), but restricted the overall cytokine expression irrespective of the individual NFAT family member ablated [this study and (14)]. Both, NFAT proteins and IRF4, enable proliferation including metabolic reprogramming of naive T cells by a shared pathway (29, 30, 47), a fact that limited T-cell expansion and ameliorated GvHD upon loss of NFATc1, NFATc2 or IRF4. Whether IRF4-deficient Tregs are equally able to preserve the GvL effect like NFAT single-deficient Tregs has to be tested next.

Cas9^+^ mice and our protocols are suitable for translational studies. Sparked by our observation that ablation of a single NFAT member in co-transplanted T cells protects like clinical calcineurin inhibition (14), we want to proceed towards translation into the clinic. Here we found that we have to take enormous care in modifying only resting human T cells, as NFAT single-deficient effector T-cells would harm the allo-HCT-receiving patient even more than non-manipulated effector T cells would.

Overall, we introduce a method to gene edit murine primary T cells by CRISPR/Cas9, in efficiency comparable to RNP transduction (3), but faster and to our opinion even easier as documented to be capable in a mixture of CD3^+^ T cells, which perform *in vivo* like naive CD3^+^ T cells derived from gene-deficient mice.

## Data Availability Statement

The original contributions presented in the study are included in the article/**Supplementary Material**. Further inquiries can be directed to the corresponding author.

## Ethics Statement

The animal study was reviewed and approved by Government of Lower Frankonia (Regierung von Unterfranken/55.2.2-2532-2-592).

## Author Contributions

SM designed and performed research as well as analyzed and discussed the data and took part in writing the manuscript. IJ, DS, and LB did experiments and analyzed data. NH and RS supported experiments. AB and AR offered resources or provided financial support and discussed the data. DM designed research and discussed data. FB-S conceptualized the research goals, acquired major funding, designed research, analyzed and discussed the data, and wrote the manuscript. All authors contributed to the article and approved the submitted version.

## Funding

This work was mainly supported by a grant from the Deutsche Forschungsgemeinschaft (DFG, German Research Foundation), project number 324392634 - TRR 221 (FB-S, AR, AB, and DM). The Fritz Thyssen Stiftung 10.13.2.215 (FB-S), the Else Kröner-Fresenius Foundation 2015_A232 (FB-S) and the DFG FOR2830 (FB-S) provided additional funding.

## Conflict of Interest

The authors declare that the research was conducted in the absence of any commercial or financial relationships that could be construed as a potential conflict of interest.
