# Supplementary material for: Rapid and Efficient Gene Editing for Direct Transplantation of Naive Murine Cas9+ T Cells
Source: Front Immunol. 2021 Jul 21;12:683631. doi: 10.3389/fimmu.2021.683631 (PMC8335400; doi:10.3389/fimmu.2021.683631)
Supplement: Supplementary file 1 [file Presentation_1.pdf]

Supplementary Material

Rapid and efficient gene editing for direct transplantation of naive murine $Cas9^+$ T cells

Snigdha Majumder[1], Isabelle Jugovic[1], Domenica Saul[2], Luisa Bell[1†], Nadine Hundhausen[1], Rishav Seal[1], Andreas Beilhack[3], Andreas Rosenwald[1,4], Dimitrios Mougiakakos[2,5], Friederike Berberich-Siebelt[1]*

[1]Institute of Pathology, University of Wuerzburg, Wuerzburg, Germany

[2]Department of Internal Medicine 5, Hematology and Oncology, Friedrich-Alexander University (FAU) of Erlangen-Nuremberg, Erlangen, Germany

[3]Department of Medicine II, Center for Interdisciplinary Clinical Research (IZKF), University Hospital Wuerzburg, Wuerzburg, Germany

[4]Comprehensive Cancer Centre Mainfranken, University of Wuerzburg, Wuerzburg, Germany

[5]Deutsches Zentrum für Immuntherapie (DZI), Friedrich-Alexander University (FAU) of Erlangen-Nuremberg, Erlangen, Germany

*** Correspondence:**
Friederike Berberich-Siebelt
path230@mail.uni-wuerzburg.de

[†] Present address: Division of Molecular and Systems Toxicology, Department of Pharmaceutical Sciences, University of Basel, Basel, Switzerland

Supplementary Table 1. List of gRNAs used

Target gene	Sequence (5' to 3')	Target domain	Target exon
mNfatc1_1	CCA GAG TGC TAT CGG TGG TC	Regulatory domain	exon 3
mNfatc1_2	CAC AGC CCC TCC GTA ACT GG	Regulatory domain	exon 3
mNfatc1_3	TAC CCG GGG TGG ACG TCT GG	Regulatory domain	exon 3
mNfatc1_4	CCG TCT CAT AGT GAG CCC TG	RSD/DBD	exon 4
mNfatc1_5	GAT GCC ATA CCT GCA CAA TG	RSD/DBD	exon 4
mNfatc1_7	GTG ACC GAA GAT ACC TGG CT	Regulatory domain	exon 3
mNfatc1_8	AGG AAC AGC TGA GAT ACC CG	Regulatory domain	exon 3
mNfatc1_9	TTC GGT CAC ACT GAC CCG AG	Regulatory domain	exon 3
mNfatc2_1	GAA GAT CGT AGG CAA CAC CA	RSD/DBD	exon 6
mNfatc2_2	CCG CCA CAT CTA CCC TAC TG	Regulatory domain	exon 3
mNfatc2_3	CCA CAG TAG GGT AGA TGT GG	Regulatory domain	exon 3
mCd90_1	ATG GCG GCA GTC CAG GCG A		
mCd90_2	CCT TGG TGT TAT TCT CAT GG		
mCd90_3	GAG CAG GAG AGC GAC GCT GA		
mCd4_1	ACT CCT AGC TGT CAC TCA A		
mCd4_2	AAG GGA AGA CGC TGG TGC T		
mCd4_3	TAA GTT TAT TGA TGA TGA G		
mCd8a_1	ATC CCA CAA CAA GAT AAC GT		
mCd8a_2	TGG GTG AGT CGA TTA TCC TG		
mCd8a_3	TGA AGC CAT ATA GAC AAC GA		
mPdcd_1	ACA GCC CAA GTG AAT GAC CA		
mPdcd_2	TGA ATG ACC AGG GTA CCT GC		
mPdcd_3	AGT TGA GCT GGC AAT CAG GG		
mIrf4_1	CCG CAT CCC GTG GAA ACA CG		exon 1
mIrf4_2	CGC ATC CCG TGG AAA CAC GC		exon 1
mIrf4_3	ACG CGT CAT GAA CTT GGA GA		exon 1
NTC Negative control crRNA IDT Cat No. 1072544			

Supplementary Table 2: Sequences of primers used for quantitative RT-PCR and Indel detection.

	**Sequence**
qRT PCR: *Nfatc1* P1 product	5' GGGAGCGGAGAAACTTTGC 3'<br>5' CAGGGTCGAGGTGACACTAGG 3'
qRT PCR: *Nfatc1* P2 product	5' GACCCGGAGTTCGACTTCGA 3'<br>5' CAGGGTCGAGGTGACACTAGG 3'
qRT PCR: *Nfatc2* product	5' GTCCCGGAGCCGCAGCCCGA 3'<br>5' AATCGAAGAGGATGGAAAAG 3'
Actb	5' GACGGCCAGGTCATCACTATTG 3'<br>5' AGGAAGGCTGGAAAAGAGCC 3'
Nfatc1 indel detection	5' TGGCAAGCTATGACCACACA 3'<br>5' TTCATGAAGGACTCACTGGGACA 3'
Nfatc2 indel detection	5' AGCATCCGTTTTAGAGGAGG 3'<br>5' GAAGGCTCTCTGATGCTGACA 3'

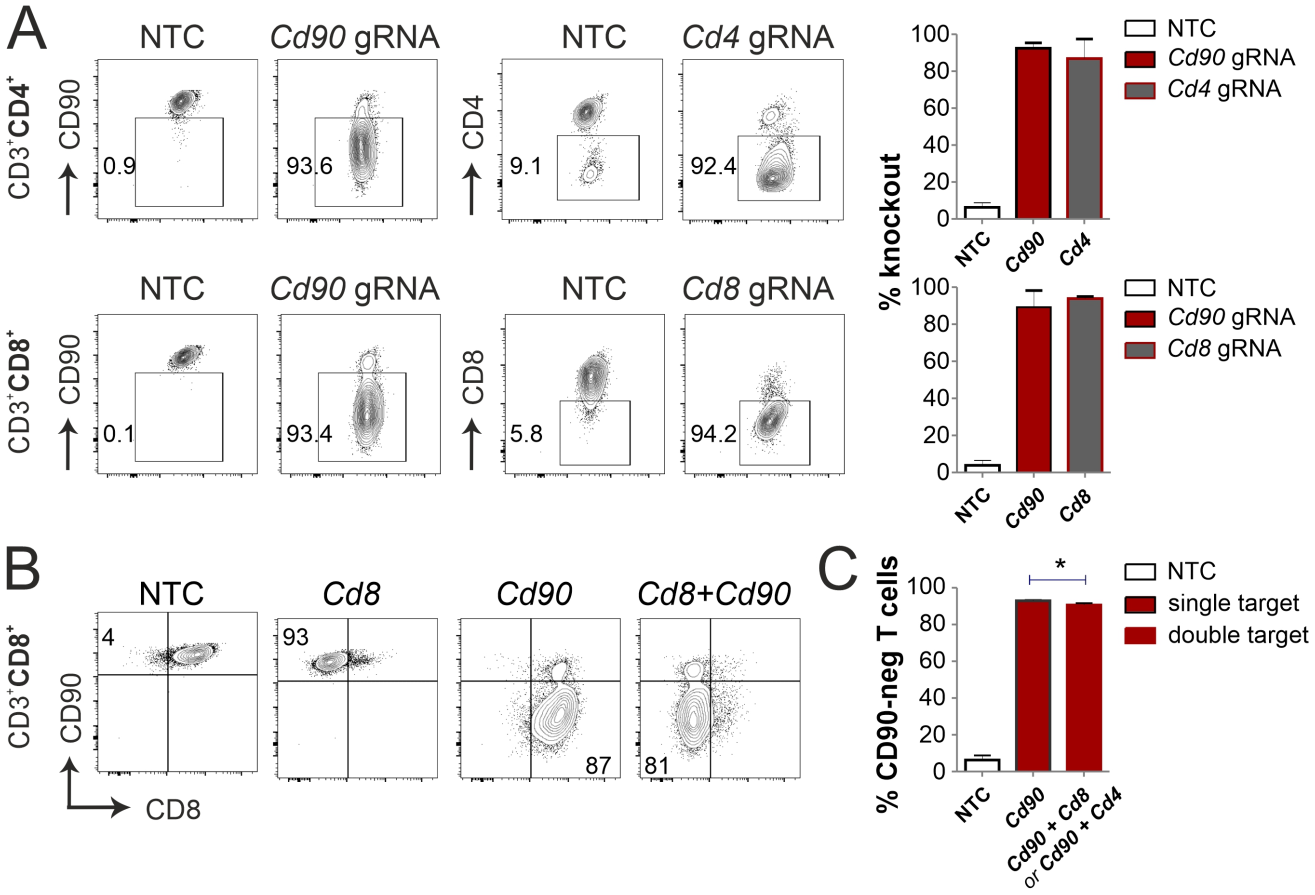

Supplementary Figure S1. gRNA-only nucleofection enables the simultaneous knockout of more than one gene in stimulated primary mouse Cas9$^+$CD3$^+$ T cells. (**A**) Analyses by surface staining and flow cytometry of CD90 and CD4 or CD90 and CD8 knockout in stimulated CD3$^+$CD4$^+$ and CD3$^+$CD8$^+$ T cells 72h post nucleofection, respectively (mean ± SD), from two independent experiments. (**B**) A flow cytometric example of simultaneous targeting of *Cd90* and *Cd8a* in CD3$^+$CD8$^+$ T cells; mean ± SD, from two independent experiments. (**C**) Cumulative evaluation of concurrent knockout of CD90 and CD4 in CD3$^+$CD4$^+$ T cells or CD90 and CD8 in CD3$^+$CD8$^+$ T cells by flow cytometry, from two independent experiments. (**A - C**) A combination of three gRNAs was used per targeted gene (Tab. S1).

A

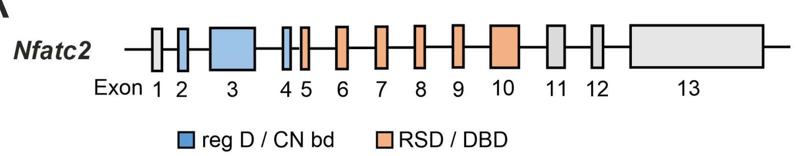

B

Nfatc2 gRNA	target exon	target domain
2,3	3	Regulatory domain
1	6	RSD / DBD

C

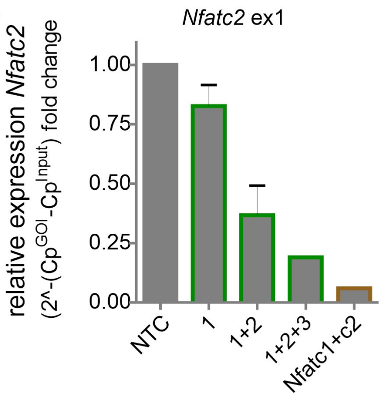

D

DNA	Sequence	clones
NTC	GAAGATCGTAGGCAACACCAAGGTCCTGG	100 %
Nfatc2 gRNA1	GAAGATCGTAGGC**C**AA**XXXXXX**GGTCCTGG	5 %
	GAAGATCGTAGGCAA**XX**CCAAGGTCCTGG	30 %
	GAAGATCGTAGGCAA**X**ACCAAGGTCCTGG	5 %
	GAAGATCGTAGGCAACACCAAGGTCCTGG	60 %
	mutational efficiency	**40 %**

E

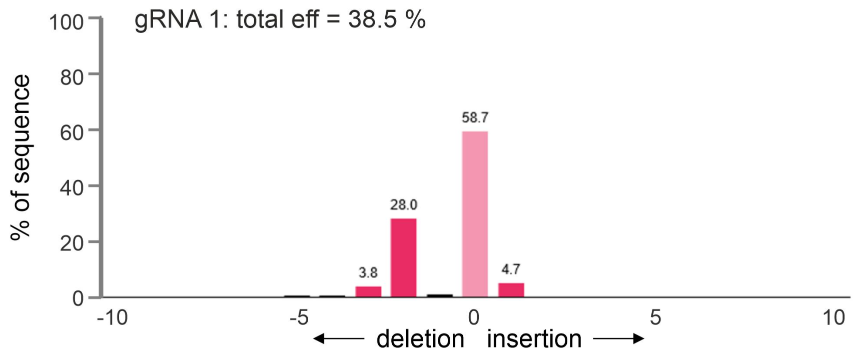

Supplementary Figure 2. NFATc2-specific gRNAs lead to Nfatc2 mutation and sufficient efficiency when combined. (**A**) Genomic structure of *Nfatc2* consists of 13 exons resulting in 12 isoforms known due to alternate splicing events. Exon 2-4 encode the regulatory domain, which includes calcineurin interaction sites. Exon 5-10 encode the Rel similarity domain, which enforces DNA binding. (**B**) Table with *Nfatc2* gRNAs and their target exons. (**C**) mRNA isolated from cells collected 72 hours post nucleofection. qRT-PCR with primers binding to exon 1 in *Nfatc2* RNA after nucleofection of one, two or three *Nfatc2*-specific gRNAs, additionally with three *Nfatc1*-specific (1+4+8) and three *Nfatc2*-specific gRNAs (1+2+3); mean ± SEM. Data are from three independent experiments. (**D**) Detection of indels in sequences of clones; established 72 h after *Nfatc2* gRNA_1 nucleofection detected by Sanger sequencing using *Nfatc2* indel detection primers. (**E**) Recognition of indels using TIDE.

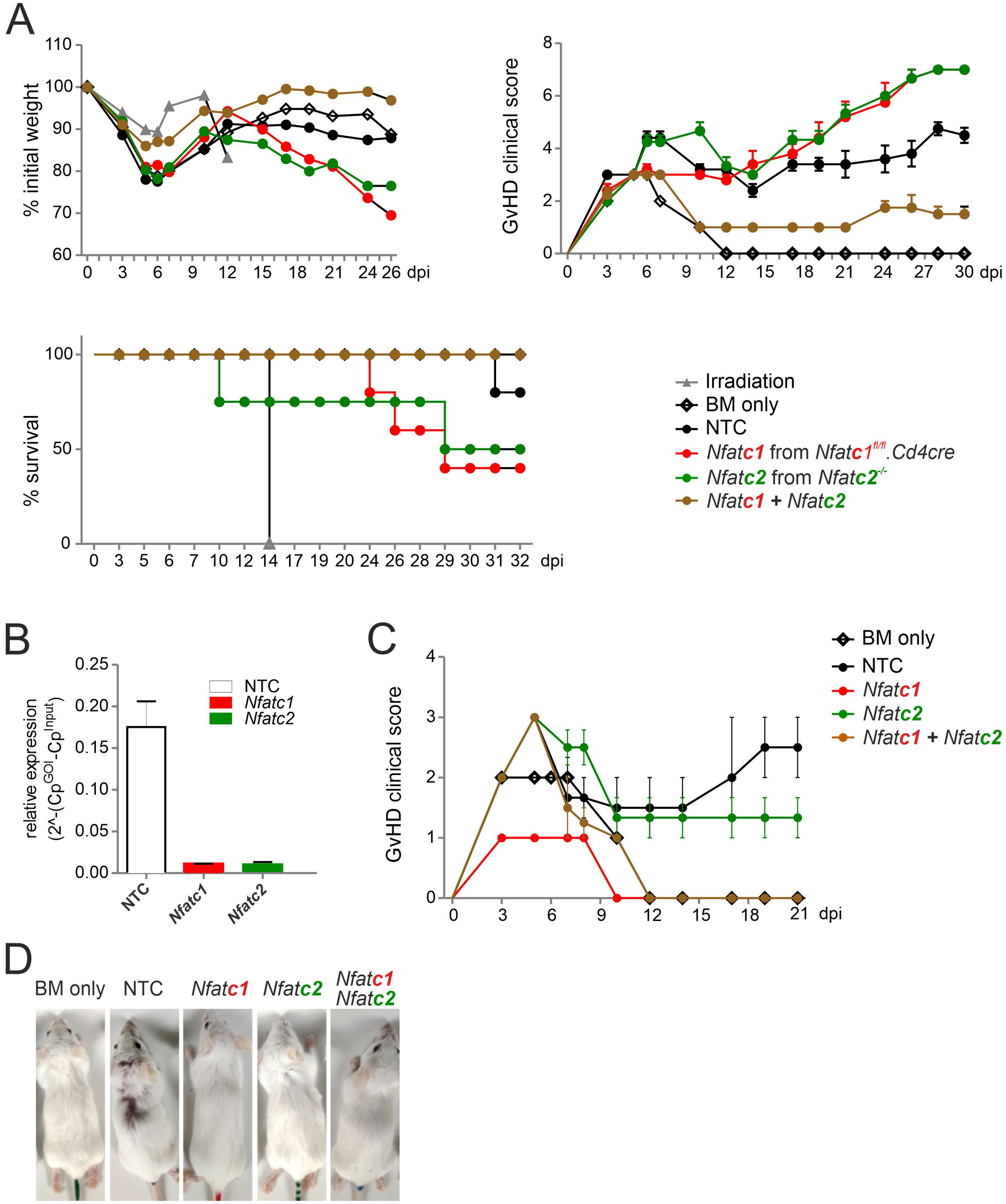
A
% initial weight
dpi
GvHD clinical score
dpi
% survival
dpi
Irradiation
BM only
NTC
Nfatc1 from Nfatc1fl/fl.Cd4cre
Nfatc2 from Nfatc2-/-
Nfatc1 + Nfatc2
B
relative expression
(2^-(CpGOI-CpInput)
NTC
Nfatc1
Nfatc2
C
GvHD clinical score
dpi
BM only
NTC
Nfatc1
Nfatc2
Nfatc1 + Nfatc2
D
BM only
NTC
Nfatc1
Nfatc2
Nfatc1
Nfatc2

Supplementary Figure 3. Pre-stimulated *Nfatc1*$^{fl/fl}$*Cd4cre* or *Nfatc2*$^{-/-}$ T cells aggravate aGVHD over time. (**A-D**) GvHD induction due to an H-2^b -> H-2^d transfer with pre-stimulated Cas9$^+$CD3$^+$ T cells. gRNA used: *Nfatc1* gRNA 4+8+9, *Nfatc2* gRNA 1+2+3. (**A**) Weight changes, GvHD clinical score and survival measured up to 32 dpi of mice transplanted with NTC or NFAT-specific gRNAs nucleofected in 3 d prestimulated Cas9$^+$CD3$^+$ T cells for *Nfatc1+Nfatc2* group or similarly treated T cells isolated from *Nfatc1*$^{fl/fl}$*.Cd4cre* or *Nfatc2*$^{-/-}$ mice for single knockout group; mean + SE; from one experiment. (**B**) qRT-PCR with NFAT-specific primers from cells stimulated *in vitro* for 3 days post nucleofection in 24 h prestimulated Cas9$^+$CD3$^+$ T cells; mean + SD; from one experiment. (**C**) GvHD clinical score measured up to 21 dpi of mice transplanted with 24 h prestimulated Cas9$^+$CD3$^+$ T cells nucleofected with NFAT gRNAs; mean ± SE; from one experiment. (**D**) Mice picture taken at 21 dpi from (**C**).

A

	IIb	4D
Cd90	14.1 %	78.2 ±1.9 %
Pdcd1	0 %	74.2 %
Nfatc1	3 %	92.55 ±3.85 %

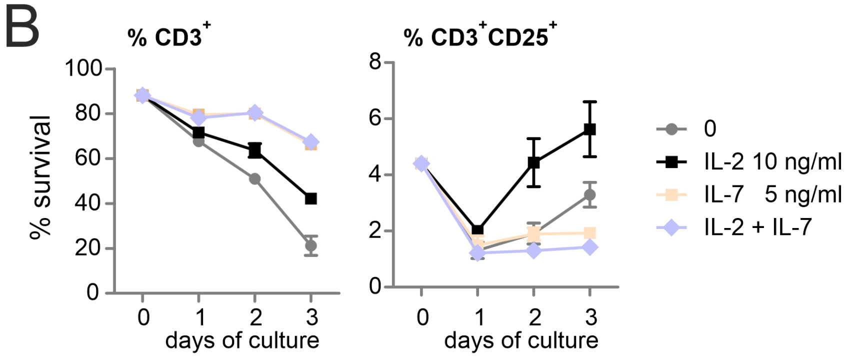

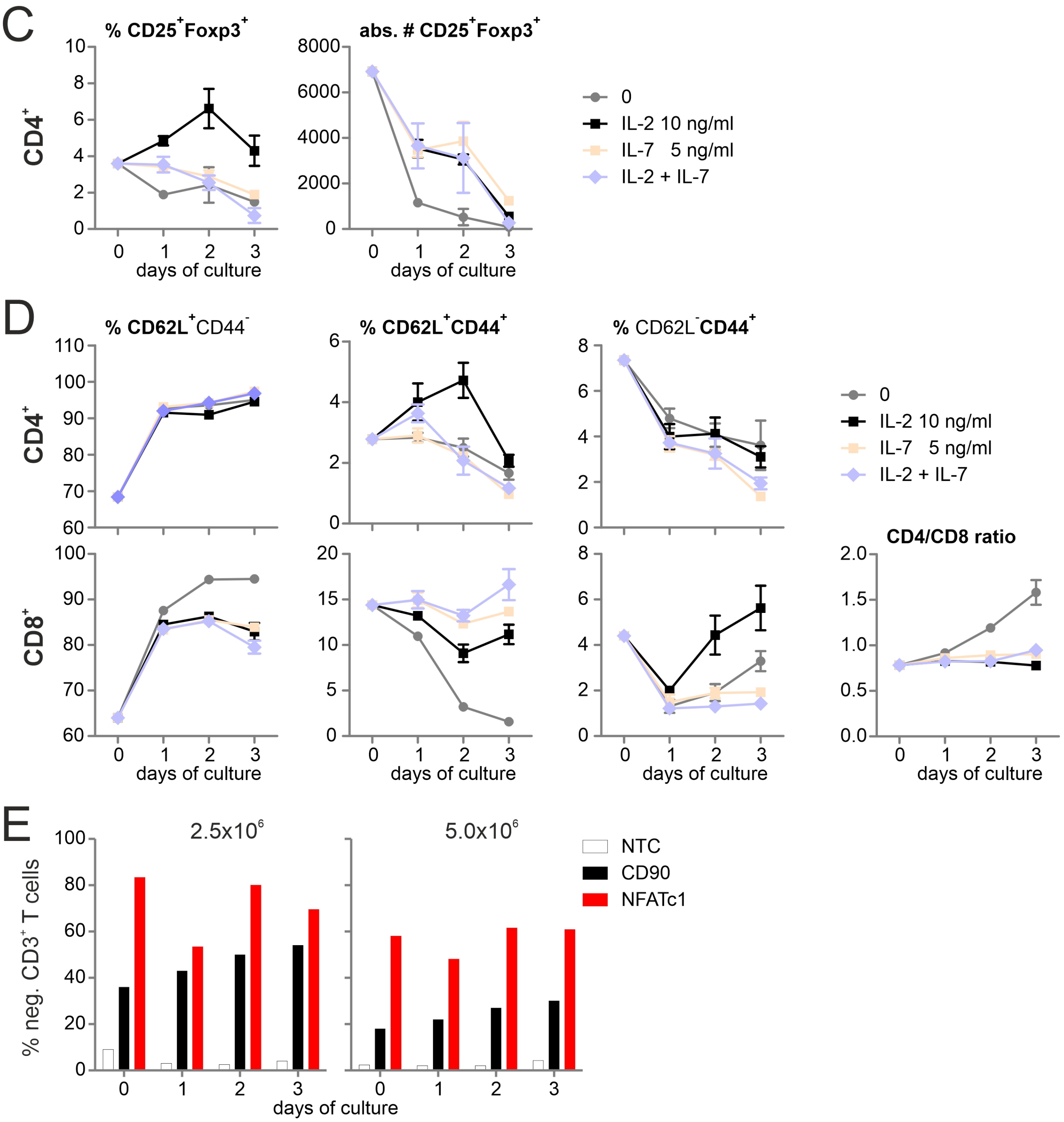

Supplementary Figure 4. Low dose IL-7 treatment after nucleofection preserves the naive status and survival the best during *in vitro*-cultures. **(A)** Table for unstimulated T cells comparing the knockout efficiencies with two versions of the electroporator. **(B-D)** Flow cytometry analysis of $Cas9^{+}CD3^{+}$ T cells nucleofected and cultured *in vitro* with/without IL-7 and/or IL-2 up to 3 d; mean ± SD. (**B**) Viability measured by Zombie live-dead staining, staining of CD3 and CD25 to measure activated T cells. (**C**) Treg percentage and absolute count by staining of CD4, CD25 and intracellular staining of Foxp3. (**D**) Frequency of $CD62L^{+}$ $CD44^{-}$ naive, $CD62L^{+}CD44^{+}$ central memory and $CD62L^{+}$ $CD44^{+}$ effector memory $CD4^{+}$ and $CD8^{+}$ T cells. CD4 to CD8 ratio was calculated from percentage. (**E**) $2.5x10^{6}$ or $5x10^{6}$ $Cas9^{+}CD3^{+}$ T cells were nucleofected with *Cd90* gRNA_2 or *Nfatc1* gRNA_4, cultured with IL-7 for indicated times and analyzed by flow cytometry after 3 d stimulation.

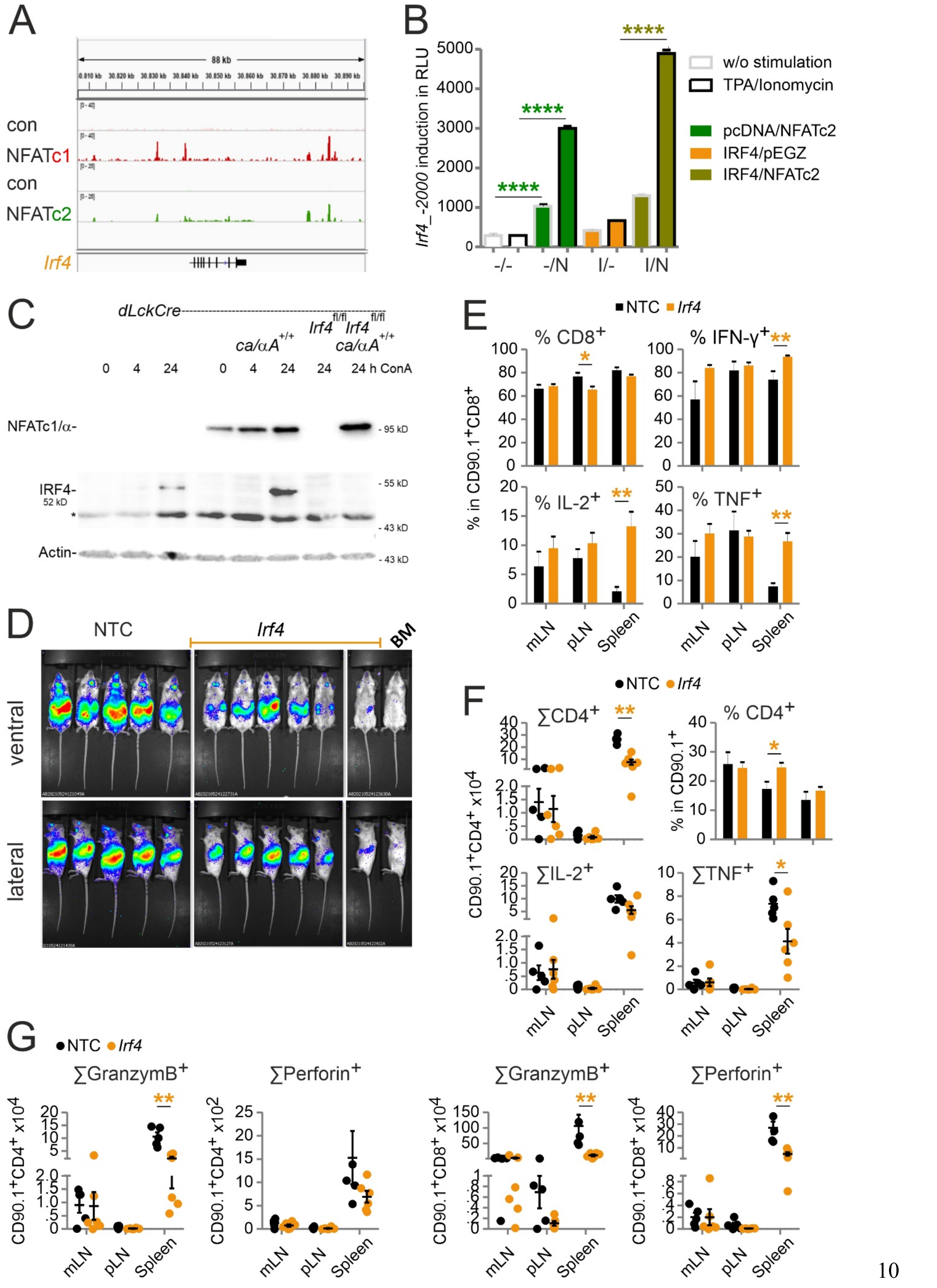
A
88 kb
con
NFATc1
con
NFATc2
Irf4
B
Irf4_-2000 induction in RLU
w/o stimulation
TPA/Ionomycin
pcDNA/NFATc2
IRF4/pEGZ
IRF4/NFATc2
-/-
-/N
I/-
I/N
C
dLckCre
ca/αA+/+
Irf4fl/fl
Irf4fl/fl ca/αA+/+
0 4 24 0 4 24 24 24 h ConA
NFATc1/α-
IRF4-
52 kD
Actin-
- 95 kD
- 55 kD
- 43 kD
- 43 kD
D
NTC
Irf4
BM
ventral
lateral
E
NTC Irf4
% CD8+
% IFN-γ+
% IL-2+
% TNF+
% in CD90.1+CD8+
mLN
pLN
Spleen
F
NTC Irf4
∑CD4+
% CD4+
∑IL-2+
∑TNF+
CD90.1+CD4+ x10^4
% in CD90.1+
G
NTC Irf4
∑GranzymB+
∑Perforin+
CD90.1+CD4+ x10^4
CD90.1+CD4+ x10^2
CD90.1+CD8+ x10^4

Supplementary Figure 5. NFAT target gene *Irf4* knockout in naïve T cells ameliorates aGvHD. (**A**) Binding of NFATc1 and NFATc2 to the *Irf4* locus. Shown are own and publicly available ChIP-seq data for CD8^{+} T cells. (**B**) Luciferase assays of *Irf4* proximal promoter (836 bp) in EL-4 cells. Cells were co-transfected with the empty vectors pEGZ and / or pcDNA (-/-), as well as constructs encoding either constitutive active NFATc2 (-/N), IRF4 (-/I) or both (N/I). Cells were left unstimulated or activated with TPA/Iono; n≥3. (**C**) *In vitro* ConA-stimulated lymphocytes collected from spleen and lymphnodes of *dLckCre* (WT), *Nfatc1^{caaA}.dLckCre* (*ca/αA$^{+/+}$*), *Irf4*$^{fl/fl}$.*dLckCre (Irf*$^{fl/fl}$) and double transgenic (*Irf4*$^{fl/fl}$.*ca/αA$^{+/+}$*) mice were analyzed by whole cell extracts and immunoblots with anti-NFATc1/α (specific for the α-peptide), anti-IRF4, and anti-Actin-β. (**D**) Ventral and lateral *in vivo* BLI at 5 dpi of mice transplanted with NTC or *Irf4* gRNA-nucleofected naïve Cas9^{+}CD3^{+} T cells. (**E-G**) Surface and intracellular *ex vivo* staining of NTC and *Irf4* gRNA-nucleofected CD90.1^{+} T cells at dpi 6; mean±SD; Mann-Whitney test (*p<0.05, **p<0.005). (**E**) Frequency of CD8^{+} in CD90.1^{+} and of IL-2, TNF and IFN-γ-positive CD8^{+} within the CD90.1^{+}CD8^{+} T cell population. (**F**) Absolute and relative numbers of CD90.1^{+}CD4^{+} T cells as well as absolute numbers of IL-2 and TNF-positive CD90.1^{+}CD4^{+} T cells. (**G**) Absolute numbers of granzymeB and perforin-positive CD90.1^{+}CD4^{+} and CD90.1^{+}CD8^{+} T cells.
